# Incorporation of Ag-doped ZnO nanorod through Graphite hybridization: Effective approach for degradation of Ciprofloxacin

**DOI:** 10.1016/j.heliyon.2023.e13130

**Published:** 2023-01-21

**Authors:** Tanu Shree Roy, Surya Akter, Monabbir Rafsan Fahim, Md. Abdul Gafur, Tahmina Ferdous

**Affiliations:** a Department of Physics, Bangladesh University of Textiles, Dhaka, Bangladesh; b Bangladesh Council of Scientific and Industrial Research, Dhaka, Bangladesh; c Department of Materials and Metallurgical Engineering, Bangladesh University of Engineering and Technology, Dhaka, Bangladesh; d Department of Textile Engineering Management, Bangladesh University of Textiles, Dhaka, Bangladesh; e Department of Physics, Jahangirnagar University, Savar Union, Bangladesh

**Keywords:** Advanced Oxidation Process, Ciprofloxacin, Degradation, Graphite hybridization, ZnO Nanorod

## Abstract

To remove the Ciprofloxacin (CIP) from aqueous solution, ZnO–Ag-Gp nanocomposite exhibited efficient photocatalytic properties. The biopersistent CIP is pervasive in surface water and also hazardous to human and animal health. This study utilized the hydrothermal technique to prepare Ag-doped ZnO hybridizing Graphite (Gp) sheet (ZnO–Ag-Gp) to degrade pharmaceuticals pollutant CIP from an aqueous medium. The structural and chemical compositions of the photocatalysts were determined by XRD, FTIR and XPS analysis. FESEM and TEM images revealed the nanorod ZnO with round shape Ag distributed on a Gp surface. The reduced bandgap of the ZnO–Ag-Gp sample enhanced the photocatalytic property which was measured by using UV–vis Spectroscopy. Dose optimization study found that 1.2 g/L is optimum for single (ZnO) and binary (ZnO-Gp and ZnO–Ag), where 0.3 g/L ternary (ZnO–Ag-Gp) exhibited maximum degradation efficiency (98%) within 60 min for 5 mg/L CIP. Pseudo 1st order reaction kinetics rate was found highest for ZnO–Ag-Gp (0.05983 min^−1^) and it decreased to 0.03428 min^−1^ for annealed sample. Removal efficiency decreased to only 90.97% at 5th run and hydroxyl radicals played a vital role to degrade CIP from aqueous solution. UV/ZnO–Ag-Gp will be a promising technique to degrade wide-ranging pharmaceutical antibiotics from the aquatic medium.

## List of abbreviations

CIP: Ciprofloxacin antibiotic
AOP: Advanced Oxidation Process
NPs: Nanoparticles
ZnO: Zinc Oxide
Ag: Silver
Gp: Graphite
ZnO–Ag: Ag-doped Zinc Oxide
ZnO-Gp: Graphite doped Zinc Oxide
ZnO–Ag-Gp: Ag-doped Zinc Oxide hybridizing Graphite

## 1 Introduction

Unconsumed antibiotics are emergent trace refractory contaminants in various natural aquatic ecosystems. These pollutants are likely to have long-term consequences for the ecosystem and people's health, such as bacterial resistance to antibiotics, hormonal disruption, and cancer tumors [1]. Fluoroquinolones (FQ) are novel class of non-steroidal antibiotics or antibacterials that are wholly artificial. They are utilized to treat infections in various body parts, eradicating or inhibiting the growth of dangerous germs. Ciprofloxacin (CIP) was a first-generation FQ introduced in the 1980s. CIP has been used to treat anthrax, TB, bladder infection, sexually transmitted illnesses, and gastrointestinal issues [2,3]. CIP has been identified as one of the top ten prioritized prescribed medicines detected in the aquatic cycle and it is a major concern for European Union programs [4]. Even a small amount of dose can promote bacterial antibiotic resistance, posing significant health and environmental dangers [5]. Because of its ecotoxicity and tendency to inspire resistance in species of bacteria, the elimination of CIP from the aquatic environment is a reason for worry [6]. High amounts of antibiotics in on-site hospital-treated wastewater may risk the microbial community and reduce future treatment efficiency [7]. It is self-evident that efficient treatment procedures for removing these medicines from the aqueous phase must be developed. Threats that the contaminants pose to the aquatic environment, efforts are being conducted to establish a process that can effectively degrade them from wastewater while still providing water [8].

Advanced Oxidation Processes (AOP) are usually utilized to break down a wide range of harmful organic contaminants [9]. AOP can be performed on the in situ creation of radical species, particularly the hydroxyl radical, superoxide radicals, and holes that enable the oxidation of micro-pollutants such as antibiotics [10–12], dyes [13], and effluents [14]. Selvakumar et al. [3] and Tran et al. [11] used photocatalysts to remove antibiotics from aqueous solutions by the AOP method and investigations were done by Klavarioti et al. [15] on the degradation of pharmaceutical pollutants from aqueous systems by AOP method. Photocatalysis based on some semiconductors is acceptable. Photons with sufficient energy are absorbed by a semiconductor, causing electrons (e^-^) to go to the conduction band while holes (h^+^) remain in the valence band (Eq. (1)). Electrons can eject to the semiconductor surface and can be captured by oxygen molecules (Eq. (2)) to emit superoxide radicals (•O_2_^−^) [11]. In parallel, organic components at the material's surface are attacked by the holes and water molecules are oxidized to form hydroxyl radicals (•OH) (Eq. (3)). The organic contaminants can be decomposed by reactive agents (i.e., •O_2_^−^, •OH and h^+^) (Eq. (4)) [12].

(1) $${Photocatalyst\;}_{\to }^{hv}{\;e}_{cb}^{-}+h_{vb}^{+}$$

(2) $${eˉ+O_{2}\;\to •O}_{2}^{-}$$

(3) h^+^ + H_2_O → H^+^ + •OH

(4) $${(•O}_{2}^{-},h^{+},•OH)+organics\;\to \;products$$

Semiconductor photocatalysis has grown in popularity and importance in the last few years as it is a better way to help the environmental rehabilitation. Choosing semiconductor materials such as ZnO, a promising photocatalyst, is an n-type semiconductor with a wide bandgap of 3.37 eV and the absorption is in the ultraviolet region of the spectrum at room temperature [8,11,13,16,17]. The physical and chemical characteristics of nanoparticles, including their utilization in various sectors, have piqued the interest of researchers. Heterogeneous photocatalysis with the help of ultraviolet radiation employing ZnO NPs is an AOP that has demonstrated remarkable ability in destroying surrounding contaminants [8,11,15]. Its photocatalytic activity is only visible under UV light and charged carrier recombination is faster than redox processes, limiting its practical applicability. Another significant disadvantage of ZnO is its photocorrosion behavior when exposed to light. For this reason the reduction of photocatalytic ability and stability [16,18], in the past few years, many researchers such as Jun et al. [19], Singhal et al. [20], Kumar et al. [13], Kim et al. [21], Reddy et al. prepared doped ZnO NPs with various dopants like Al, Ga, Cu, Ag, Al–Ag, Co–Ag with multiple methods. The researchers investigated the effects of dopants on the properties of doped ZnO nanoparticles. Kumar et al. synthesized Ag-doped ZnO NPs to study the effect of silver (Ag) on ZnO NPs properties and photocatalytic utilization. They revealed that Ag-doped ZnO NPs degrade methylene blue more efficiently than pure ZnO NPs.

Silver has been recognized as a promising candidate for shallow acceptor fabrication [13]. Improvement of surface charge distribution, conduction band acceptance developed through photoreaction when exposed to UV light and discarding of photogenerated electron-hole recombination are all the qualities of Ag-doped in ZnO [21]. Shah et al. [17] investigated magnetic, optical, and structural applications on Ag-doped ZnO NPs, and Bhaviya Raj et al. [16] exhibited the antibacterial property of Ag-doped ZnO nanocomposites and photocatalytic activity towards anionic and cationic dyes. They synthesized Zinc oxide/Silver nanocomposites and established the effect of the degradation of methyl orange, methylene blue, and crystal violet. With six recycles, Peng et al. explored the ZnO nanoparticles on the reduced graphene oxide surface to see whether they might increase their photocatalytic activity and stability [22]. Chen et al. [23] investigated that the core-shell of carbon-coated ZnO can enhance the absorption and optical properties, removing methyl orange.

The present study used the hydrothermal method to synthesize pure ZnO, Ag-doped ZnO NPs, Graphite (Gp) doped ZnO NPs, and Ag-doped ZnO NPs hybridizing with graphite. These samples were prepared before and after annealing. The Ag particles and Gp sheets can operate as storages for electrons by decreasing the recombination of photogenerated charge carriers. The carbon layer functions as a promoter which makes the behavior of the scattered particles more steady [24]. According to Tran et al., the addition of Ag reduced the rate of electron-hole pair recombination and increased photocatalytic activity across a wide range of light wavelengths. The graphite surface also worked as an electron sink to display ZnO photocorrosion. Raw graphite was chosen for its exceptional qualities, including thermal stability, resistance to corrosion, electron trap-ability, and cost-effectiveness [24]. To accomplish the combination of these remarkable qualities, we have used the hydrothermal method to synthesize Ag-doped ZnO NPs on the graphite surface (ZnO–Ag-Gp composite) to decompose CIP from an aqueous medium.

Noticeably, until now, most prior studies focused on Ciprofloxacin's photodegradation with Gd2WO6 loaded ZnO/bentonite nanocomposite, Z-scheme multi-shelled ZnO/AgVO3 spheres, Z-scheme ZnO/Ag/Ag_3_PO_4_ and N–ZnO/CdS/Graphene oxide composite [3,[25], [26], [27]], while investigations towards the degradation of CIP by the combination of Ag, Gp onto ZnO are still new approaches according to our knowledge. In the present study, the ZnO–Ag-Gp composite exhibited more efficient photodegradation of CIP than the Z-scheme ZnO/AgVO3 spheres, and ZnO/Ag/Ag_3_PO_4_ composite. In this research, we examine the facile hydrothermal synthesis of silver and graphite doped onto ZnO to establish a brand-new idea that the ZnO–Ag-Gp composite has the significant photocatalytic property of degradation of CIP from aqueous medium. According to authors best knowledge, there was no similar literature on the doping of silver in ZnO, hybrid graphite that influenced the degradation of CIP from the aquatic medium. The synthesis process was cost-effective because no extra chemical was used except sodium hydroxide and ethanol and the low-temperature facile hydrothermal technique was applied. This research established and analyzed the idea of the degradation of CIP before and after the annealing treatment of the prepared nanocomposites that improvised the outlook on a better optimum process to implement on large industrial scale. The present study gave away that the efficiency of degradation of CIP was more prominent in before annealed samples than after annealed samples. According to the comparative investigation on before and after annealed, this was another achievement of cost-effectiveness and simplicity.

The pure and doped ZnO NPs were synthesized and characterized via different techniques such as X-ray Diffraction (XRD), Field Emission Scanning Electron Microscopy (FESEM), Transmission Electron Microscopy (TEM), Selected Area Electron Diffraction (SAED), X-ray Photoelectron Spectroscopy (XPS), Fourier-Transform Infrared (FTIR) analysis and UV–visible Spectroscopy to analyze various structural, morphological, chemical and optical properties. The photocatalytic research has been conducted by applying the AOP to investigate of degradation of CIP from the water.

## 2 Materials and methods

### 2.1 Materials

Zinc acetate dihydrate (99% is reagent grade from Scharlau, Germany), Ethanol, Sodium hydroxide, Graphite, Silver nitrate (99.9% Merck KGaA, Darmstadt, Germany) were purchased from S. A. Scientific. Ciprofloxacin (CIP) was from Sigma-Aldrich, Chemie GmbH, Kappelwegl, Germany and it is certified in accordance with ISO/IEC 17025 and ISO 17034 standard. No extra process is done to enhance purity as the preparation was done using materials of highest purity. Isopropyl Alcohol (IPA) (99.8% Merck KGaA, Darmstadt, Germany), Ethylene-diaminetetra-acetic acid disodium salt dihydrate (EDTA-2Na) (99.9% Merck KGaA, Darmstadt, Germany), Acrylamide (99.9% Merck KGaA, Darmstadt, Germany), Deionized water was collected from an ISO certified lab (INARS, BCSIR, Dhaka, Bangladesh).

### 2.2 Synthesis of ZnO/ZnO–Ag/ZnO-Gp/ZnO–Ag-Gp composites

**Fig. 1:**
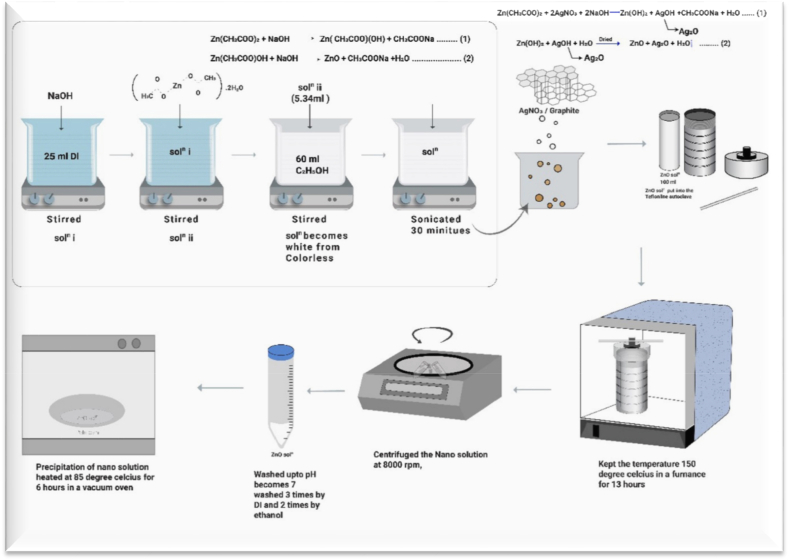
Schematically, the synthesis steps of ZnO, ZnO–Ag, ZnO-Gp, and ZnO–Ag-Gp nanocomposites prepared by hydrothermal method.

ZnO Nps were synthesized using a low-temperature autoclave with 100 ml internal volume and materials were Zinc acetate dihydrate (1 M), Ethanol (60 ml), Sodium hydroxide (NaOH) (10 M), Deionized water (25 ml). The precursor was prepared by dissolving Zn(CH_3_COO)_2_·2H_2_O and NaOH in 25 ml of deionized water, as shown in Fig. 1. Firstly the aqueous solution of NaOH was prepared (sol^n^ i), then Zn(CH_3_COO)_2_·2H_2_O was added to the solution (sol^n^ i) with continuous stirring. 5.45 ml of precursor solution was mixed with 60 ml of ethanol in a beaker under constant stirring. The solution (sol^n^ ii) turned from colorless to white. The mixed solution was pre-treated in an ultrasonic water bath for 20 min before being transferred to a Teflon-lined autoclave. The autoclave temperature was 150 °C and held for 13 h in a furnace [28]. The autoclave was then rested at room temperature and removed from the furnace. The precipitation was collected by centrifuging at 8000 rpm and washed several times with DI water and ethanol. The washing process was repeated until the pH of the solution was 7. According to the following reactions Eq. (5) & Eq. (6), ZnO nanopowders were synthesized and the sodium salt (CH_3_COONa) was eliminated by washing the sample several times with ethanol and deionized water [29]. Finally, the product was dried in a vacuum oven at 85 °C for 6 h to obtain ZnO nanopowder.

(5) Zn(CH_3_COO)_2_ + NaOH → Zn(CH_3_COO)(OH) + CH_3_COONa

(6) Zn(CH_3_COO)(OH) + NaOH → ZnO + CH_3_COONa + H_2_O

The Ag-doped ZnO (ZnO–Ag) was prepared by a similar procedure. Only 0.2% Silver nitrate of the precursor was added to the solution. The ZnO–Ag-Gp and ZnO-Gp were prepared by similar methods; only the graphite was taken for 15% of the precursor. Half of the collected powders were annealed at 500 °C for 2 h.

During synthesis between zinc acetate [Zn(CH_3_COO)_2_·2H_2_O] and silver nitrate (AgNO_3_) with deionized water (H_2_O) in equation (7), sodium hydroxide solution (NaOH) was used as a precipitating agent to form Zn(OH)_2_, AgOH, CH_3_COONa, 2 parts Sodium Nitrate (NaNO_3_) and H_2_O. The precipitation was washed with deionized water to form Zn(OH)_2_, AgOH and H_2_O. Again the precipitation was washed with ethanol to form Zn(OH)_2_ and AgOH. In equation (8), the Ag_2_O doped ZnO nanocomposite was synthesized by dried at 85 °C to form Zn–*O*–Ag bonding [30].

(7) Zn(CH_3_COO)_2_ + 2AgNO_3_ + 2NaOH → Zn(OH)_2_ + AgOH + CH_3_COONa + H_2_O → Ag_2_O

The precipitation was washed with DI three times and three times with Ethanol.

(8) Zn(OH)_2_ + AgOH + H_2_O → ZnO + Ag_2_O + H_2_O↑

The produced nanopowder and nanosolution are shown in Figure S2.

### 2.3 Characterization methods

The X-ray diffraction method was used to analyze the structure of crystalline phases present in the material and thereby disclosed chemical composition. Bruker D8 diffractometer, Cu-Kα, λ = 1.5418 Å, target voltage 40 KV and current 40 mA, Bruker GmbH, German was used to obtain the XRD patterns of the prepared catalysts. The average crystallite size (D) was determined using Debye-Scherrer's formula (Eq. (9)).

(9) $$D=\frac{k\lambda }{\beta \;\cos\;\theta }$$

An FTIR Spectrometer, Shimadzu IR Tracer, Japan, collected high-resolution spectral data over the 400-4000 cm^−1^ wavenumber range. To obtain statistics about the surface morphology and composition, Field Emission Scanning Electron Microscopy (FESEM) EV018, ZEOL, Japan, scanned a focused electron beam over a surface to produce an image. The reactor containing a 24-W UV lamp with λ = 254 nm and path length of 4.5 cm, Kappelwegl, Germany, was used to provide UV radiation for photodegradation. UV–vis spectrum was determined by UV 1601 Spectrophotometer, SHIMADZU, Japan, and the wavelength range was used between 200 and 800 nm. The Transmission electron microscopy (TEM), Talos F200× Thermo Fisher Scientific, USA, was used. The sample was prepared using carbon-coated copper grade (200 mesh), 200 kV to operate the TEM image. The powder of the sample was dispersed in ethanol by sonication for 30 min before the drop on copper grade and dried copper grade overnight for testing. The Ultrasonic Cleaner, Model VGT-1860QTD, frequency-40KHz, China, was used for sonication. To identify the chemical state of elements, K-Alpha X-ray Photoelectron Spectrophotometer (XPS), Thermo Fisher Scientific, UK, was used. The Avantage software was utilized for Surface analysis in the XPS investigation.

### 2.4 Photodegradation experiments

The molecular formula of Ciprofloxacin is C_17_H_18_FN_3_O, with a molecular mass of 331.347 g mol^−1^ [31]. The CIP was degraded by composites conducted under UV radiation. The reactor contains three lamps, each with an 8-Watt UV lamp with a wavelength of 254 nm and the distance between the solution and the lamp is 4.5 cm which was explained by the experimental set-up represented in our previous work [32]. The structure of CIP has been included in the Supplementary Information.

In this research, the synthesized catalysts ZnO, ZnO–Ag, ZnO-Gp and ZnO–Ag-Gp had distributed in a 100 ml CIP (5 mg/L) aqueous solution at dosages of 0.1 g/L, 0.3 g/L, 0.6 g/L, and 1.2 g/L for each. A UV–visible spectrophotometer was used to quantify the antibiotic's concentration level by measuring its absorption at 278 nm. The volume of the liquid is 100 ml and the depth is 4 cm from the UV light to the surface of the liquid. Also, the surface area comes to 41.87 cm^2^. The solution was agitated with a magnetic stirrer in the dark for 60 min before exposing UV light to establish adsorption-desorption equilibrium. 5 mL of samples were obtained at specified intervals and rested for 24 h at room temperature to settle the precipitation. Then the supernatant was filtered with a 0.22-μm 25 mm PTFE nylon membrane filter to remove suspended particles. After 24 h, the concentration of CIP in the sample was measured. The degradation of CIP using ZnO–Ag-Gp samples was tested for five consecutive runs. ZnO samples were tested for four runs to observe the stability of the photocatalyst for large-scale applications. The active species trapping test was performed with the addition of different scavengers like Acrylamide, Isopropyl Alcohol (IPA), Ethylene-diaminetetra-acetic acid disodium salt dihydrate (EDTA-2Na).

The Pseudo 1st order formula (Eq. (10)) was used to determine the kinetics of CIP deterioration [11,33].

(10) $$\ln\left(\frac{C_{t}}{C_{0}}\right)=k_{obs}t$$

where k_obs_ is the observed rate constant, where Cₒ is the initial concentration of the CIP and C_t_ is the concentration of the CIP having photo-irradiation. The k_obs_ value is obtained by plotting –ln(C_t_/C_0_) vs. reaction time (t) accepts a straight line with a slope equal to k_obs_ [11].

The removal efficiency (Eq. (11)) was determined by

(11) $$removal\;\left(\%\right)=\frac{C_{0}-C_{t}}{C_{0}}\times 100\;\%$$

## 3 Results and discussion

### 3.1 Structure, morphological and elemental analysis of synthesized photocatalysts

**Fig. 2:**
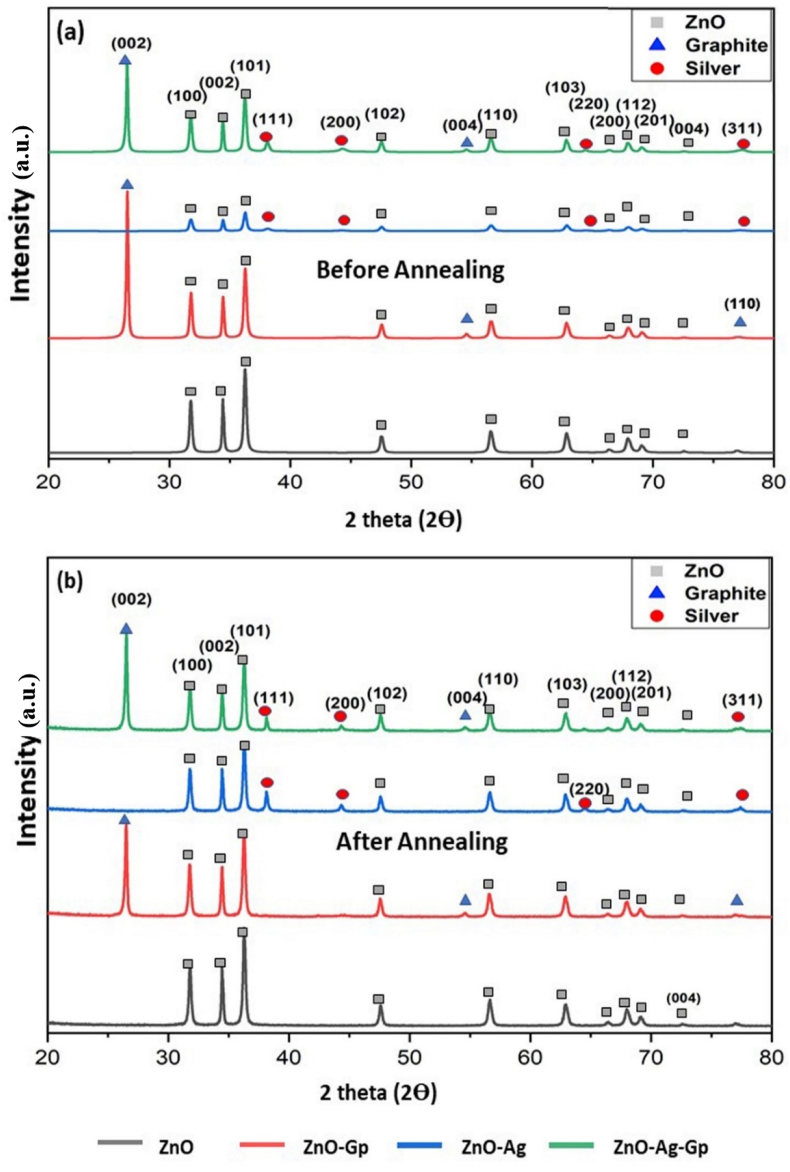
X-ray powder diffraction patterns of ZnO, ZnO-Gp, ZnO–Ag, and ZnO–Ag-Gp nanocomposites (a) before and (b) after annealed.

Fig. 2(a) and (b) showed the XRD patterns of as-prepared and annealed ZnO samples. The peak and relative intensities of the diffraction lines positively matched with standard hexagonal ZnO (JCPDS no-01-080-0074) diffraction data for different planes (100), (002), (101), (102), (110), (103), (200), (112), (201), (004), (202) indexed with the 2θ values of 32.7743°, 34.4354°, 36.2623°, 47.5607°, 56.6111°, 62.8808°, 66.4029°, 67.9723°,72.5788°, and 76.9868°. This indicates the formation of ZnO nanoparticles from the precursor with near certainty [34].

The lattice constants of the ZnO were found as a = b = 3.2490 Å and c = 5.2046 Å. In the diffraction patterns, no additional peak of any other phase, such as Zn(OH)_2_ or remaining sodium compounds, was observed, confirming the excellent purity of the produced ZnO products [35]. Diffraction lines of the prepared ZnO, ZnO–Ag, ZnO-Gp and ZnO–Ag-Gp nanocomposite were also relatively sharp, suggesting a high crystallinity for all samples. The average crystallite size of all the samples was calculated using the most prominent peaks, namely- (100), (002), and (101).

For ZnO–Ag, the positions and relative peak intensities of ZnO and Ag planes were flawlessly toned with standard data in Fig. 2. Matches were found of the (111), (200), (220), and (311) planes with 2 theta values of 38.1258°, 44.2462°, 63.6667°, and 77.2353° of the face-centered cubic structure of Ag with JCPDS no- 01-087-0717. The lattice constants of Ag were a = b = c = 4.085 Å. The average crystallite size of the ZnO for (100), (002), and (101) planes was calculated to be 30.04 nm, which was reduced with the addition of Ag to 25.97 nm for ZnO–Ag (Table 1), according to the peak of Ag was comparatively small because only 0.2% of AgNO_3_ was added to the precursor.

**Table 1:** Comparative study of FWHM and average crystallite size of catalysts before and after annealing with (100), (002), (101) planes.

		FWHM		d-spacing		Crystallite size		Avg. Crystallite size in nm	
Catalysts	hkl	before annealing	after annealing	before annealing	after annealing	before annealing	after annealing	before annealing	after annealing
ZnO	(100)	0.2690	0.2647	2.8139	2.8112	28.4046	28.8616	30.0474	30.5587
	(002)	0.2086	0.2106	2.6023	2.5991	36.3737	36.0240		
	(101)	0.2977	0.2818	2.4753	2.4730	25.3639	26.7906		
ZnO-Gp	(100)	0.2768	0.2754	2.8128	2.8130	27.6043	27.7468	29.1767	29.5164
	(002)	0.2151	0.2180	2.6017	2.6007	35.2804	34.8082		
	(101)	0.3063	0.2904	2.4746	2.4744	24.6453	25.9944		
ZnO–Ag	(100)	0.3330	0.2606	2.8134	2.8112	22.9444	29.3201	25.9709	31.1217
	(002)	0.2291	0.2044	2.6019	2.5992	33.1173	37.1130		
	(101)	0.3455	0.2803	2.4751	2.4729	21.8511	26.9320		
ZnO–Ag-Gp	(100)	0.2811	0.2664	2.8146	2.8114	27.1824	28.6840	30.2321	30.6591
	(002)	0.1967	0.2058	2.6029	2.5996	38.5808	36.8773		
	(101)	0.3028	0.2858	2.4759	2.4732	24.9332	26.4159		

Kayani et al. [36] agreed that Ag separation on the grain boundary of ZnO prevented grain development, resulting in reduced crystallite size when Ag was doped on ZnO. The XRD graphs of undoped ZnO and ZnO–Ag in Fig. 2 show that the intensity of the diffraction peaks widened and reduced as the Ag content was doped. Sagadevan et al. [30] suggested a similar result when Ag ions were substituted for the ZnO host lattice size. The calculated crystallite size of the Ag in ZnO–Ag was 7 nm. The intensity of the peak of the Ag was comparatively small because only 0.2% of AgNO_3_ was added to the precursor.

From the graph of the ZnO-Gp, the resemblance with the diffraction planes- (002), (004), (110) with 2θ values of 26.5169°, 54.5819° and 77.0213° confirms the presence of a hexagonal structure of graphite phase of JCPDS no-00-008-0415. Graphite has lattice constants a = b = 2.4741 Å and c = 6.72 Å for ZnO-Gp composite. The average crystallite size of ZnO for ZnO-Gp NPs was calculated as 29.17 nm compared to the 30.04 nm of undoped ZnO and the crystallite size of Gp was 28.32 nm. For ZnO–Ag-Gp, there were significant peaks for ZnO, Graphite, and Ag (cubic). Fig. 2 revealed the presence of Ag with the resembling planes (111), (200), (220), and (311). In addition, two prominent peaks (002) and (004) were recognized in the graphite structure. The calculated crystallite size of the Ag and Gp in ZnO–Ag-Gp was 14.12 nm and 28.24 nm, respectively. They did not observe any silver (Ag) or graphite (Gr) peaks in their corresponding XRD patterns. In this current study, the results revealed that the crystalline structure of the graphite material and silver were intact and their pristine nature was preserved. The investigation through FESEM and EDX recognized all the elements’ existence separately and clarified the presence of particle size in the nano range for pure and doped nanocomposites. The crystallite sizes were investigated to rise slightly after the annealed treatment [35]. The intensity of the peaks increased and the Full Width at Half Maximum (FWHM) decreased, with annealing, suggesting improvement in the crystallinity of the prepared samples [35,37,38]. There is no significant transformation in diffraction peaks between before and after annealing nanostructures except scant variation in crystallite size of the significant peaks. The effect of annealing on the structures of the NPs was relatively insignificant according to the XRD patterns, except for a somewhat increase in crystallinity with the samples having Ag (i.e. ZnO–Ag and ZnO–Ag-Gp). After finding the structural ratification, FTIR spectroscopy analysis (Supplementary Information Figure S3) included the chemical and functional bonding of before and after annealing samples.

**Fig. 3:**
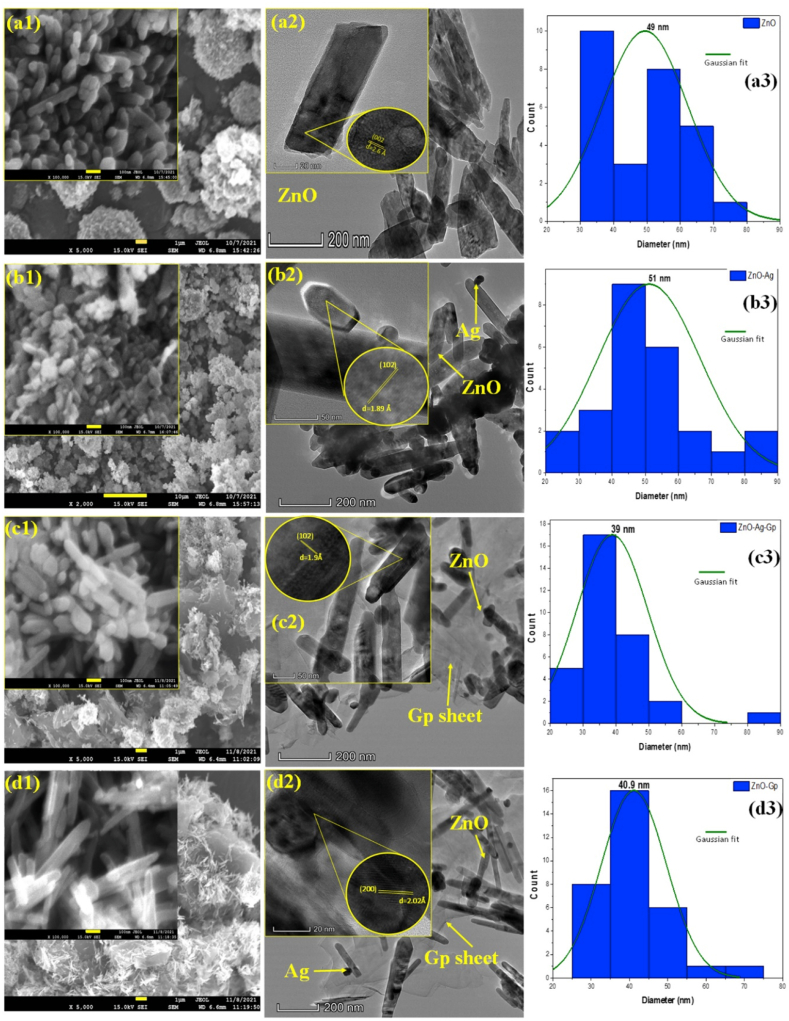
FESEM images of (a1), ZnO (b1), ZnO–Ag (c1) ZnO-Gp, (d1) ZnO–Ag-Gp NPs (inset high magnification, scale 100 nm),TEM images of (a2) ZnO (b2), ZnO–Ag (c2) ZnO-Gp, (d2) ZnO–Ag-Gp NPs (inset high magnification), Gaussian distribution of), (a3) ZnO (b3), ZnO–Ag (c3) ZnO-Gp, (d3) ZnO–Ag-Gp NPs.

The surface morphology of ZnO, ZnO–Ag, ZnO-Gp, and ZnO–Ag-Gp composites with different magnifications are shown in Fig. 3. The shape of the ZnO particle at high magnification of scale was a nanorod which was almost similar to the investigation of Wei et al. [28]. A cotton ball-like Ag nanoparticle is shown in Figure S4 [39]. With the addition of Ag/ Gp and Ag-Gp altogether, it becomes more elongated. The ZnO nanoparticles formed over the graphite surface were nanorod-shaped [40].

According to EDX analysis, it was observed that all the particles were present significantly in the composites as shown in Figure S5 (Supplementary file). The constitution of each compound was determined using EDX spectra and the Zn, O, Ag, and Carbon (C) were detected in all doped samples respectively (Table S2), with addition of the successful synthesis followed by the hydrothermal technique. The atomic ratio of Zn:O is about 1.68, which satisfied the previous report [41]. The ratios of Ag:Zn and Gp:Zn are 0.053 and 2.4 respectively, those agree with the incorporated atomic ratio [16,34,41]. Table S2 identifies that the weight and atomic percentage(%) of Zn and O are reduced when Ag and Gp are added to the composite formation [42].

TEM imaging was used to better understand the produced nanocomposites' shape, particle size, and crystallinity. The TEM images in Fig. 3 (a2,b2,c2,d2) revealed nearly identical morphological structures for the undoped and doped ZnO samples, showing that doping did not affect the ZnO sample's morphology. Fig. 3 (a2) demonstrates that the nanorod shape of ZnO with an average diameter of 49 nm was investigated with a gaussian histogram in Fig. 3 (a3) and the length is 221 nm for ZnO. The diameter and length of the nanorod are 51 nm and 271 nm, respectively. The aspect ratio of 4.51 for ZnO NPs was increased to 5.31 for the ZnO–Ag nanocomposite. This is the indication of the incorporation of Ag onto the ZnO Nps. Images obtained from the TEM examination evident of the production of ZnO nanoparticles deposited on the surface. The Ag nanoparticle focused like a round (ball) shape which is mainly demonstrated in Figure S4 of the supplementery file.

The diameter and length of the ZnO nanorod were 40.9 nm and 202 nm respectively for the ZnO-Gp nanocomposite. The TEM image in Fig. 3 (d2) indicates the coexistence of ZnO NPs, Ag NPs, and Gp sheets in the composite structure very significantly. The ZnO nanorods are randomly distributed on Gp sheets in low magnification. Similar observations were revealed by Khurshid et al. [43]. The ZnO nanorods are identified very significantly from the high magnification inset images (Fig. 3 (a2, b2, c2, d2)). The determining diameter and the length of the nanorods are about 39 nm and 351 nm, respectively, for the ZnO–Ag-Gp composite. The aspect ratio of 5.31 of ZnO–Ag was increased to 9 for the ZnO–Ag-Gp nanocomposite. The reason may be for forming a new nucleation site due to the Gp sheet and the nanorod becomes elongated. The AgNPs have approached to distribute on the ZnO nanorod. These NPs with the increased surface-to-volume ratios can contribute to the increased photocatalytic activity because of their highest photon absorption capacity. In Fig. 3 (a2,b2,c2,d2), the high-resolution TEM images demonstrate the inter-planar spacing of 2.6 Å, 1.89 Å,1.9 Å, and 2.02 Å for ZnO, ZnO–Ag, ZnO-Gp, and ZnO–Ag-Gp samples, respectively, which could be matched well with (002), (102), (102), and (200) crystal planes in XRD analysis.

The single-crystalline [44] nature of the produced ZnO nanorod was confirmed by the SAED pattern shown in Figure S6 (a) of the supplementery file. (200) planes were matched with the d-spacing of the XRD analysis of hexagonal ZnO. The sharp diffraction rings by the SAED patterns (Figure S6 (b,c, and d)) depicted the polycrystalline [45] properties of the nanoparticles. From the SAED analysis, (111), (102), and (200) planes of ZnO in ZnO–Ag, ZnO-Gp, and ZnO–Ag-Gp respectively were obtained which satisfied XRD results (Complete Figure S6 in Supplementery file).

### 3.2 Element compositions with chemical states and thermal stability analysis

**Fig. 4:**
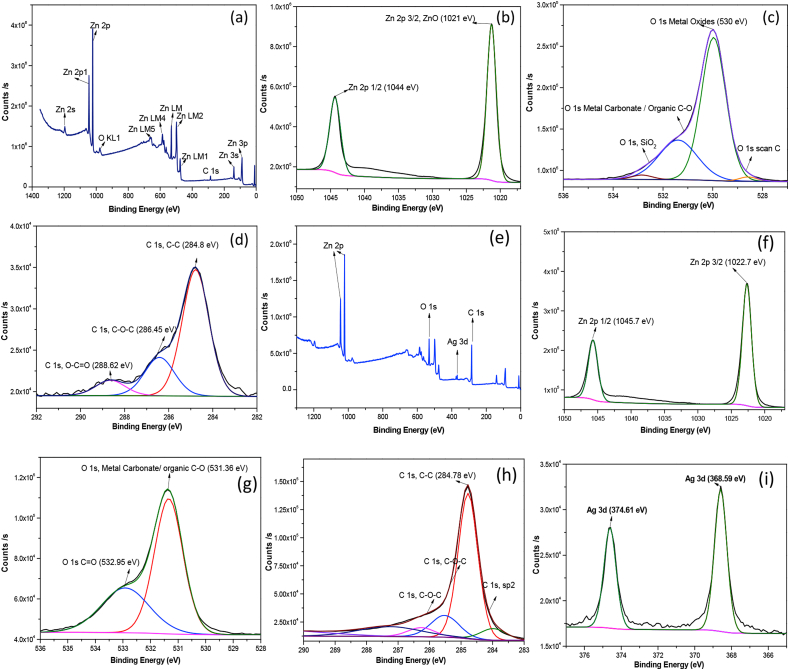
XPS full spectra of (a) pure ZnO,(e) ZnO–Ag-Gp XPS, narrow spectra of (b) Zn 2p for ZnO, (f) Zn 2p for ZnO–Ag-Gp (c) O 1s for ZnO, (g) O 1s for ZnO–Ag-Gp,(d) C 1s for ZnO,(h) C 1s for ZnO–Ag-Gp and (i) Ag 3d for ZnO–Ag-Gp sample.

XPS spectra of the pure ZnO (Fig. 4(a)) is indicating the purity of the synthesized sample displaying the peaks of Zn, O and C atoms. The pair of peaks at 1021 eV and 1044 eV correspond to the Zn 2p3/2 and Zn 2p1/2 respectively (Table 2) which proves that Zn is in the ZnO form [11]. The high spin-orbit interaction caused the splitting of Zn-2p (Fig. 4(b)) states to be around 23 eV [46]. The Zn 2p3/2 occurred at 1022.1 eV, Zn 2p1/2 at 1045.1 eV, according to a previously published report of XPS results of stoichiometric ZnO which can be explained by the charge transfer from Zn^2+^ to O^2−^ due to vacancies [47]. The O 1s peak (Fig. 4(c)) of the present oxygen is observed at 530.5 eV. As an internal reference, the binding energy of the C 1s peak of unintended carbon (C) at 285.8 eV was used, which corresponds to the C–O bond [3,11]. The presence of carbon in the sample was most likely owing to acetate vestige [46,48].

**Table 2:** Binding energy (eV) of Zn 2p, O 1s, C 1s, and Ag 3d of the prepared samples.

Sample	Zn 2p1/2	Zn 2p3/2	O 1s	C 1s	Ag 3d 3/2	Ag 3d 5/2
ZnO	1044	1021	530.5	285		
ZnO–Ag-Gp	1045.7	1022.7	531.27	284.38	374.61	368.59

In the XPS spectra of the ZnO–Ag-Gp composite, similar peak positions of Zn 2p and O 1s are observed (Fig. 4(e–h)). As a result of the charge transfer in the binding energy of the XPS spectrum, it can be determined that the Zn-2p peak shift in doped samples versus pure samples suggests that oxygen deficit is the dominating factor versus Zn deficiency [47].

According to Fig. 4(g), it was observed that the O 1s curve was asymmetric. Two different types of oxygen groups in the samples were identified in the lower binding energy involved to O^2−^ ions of Zn − O bonding for crystal. Another peak was activated in the hydroxyl group due to structural defects [46]. These might play a prominent part in photocatalytic activity because they protect from the rejoining of electron hole pairs. Ag-doped ZnO showed a shift in the O 1s spectrum to the lower binding energy. The peaks at 368.59 eV and 374.61 eV (Fig. 4(i)) for the composite sample represent the Ag 3d5/2 and Ag 3d3/2, respectively, which indicates a shift in the lower binding energy of AgNPs compared to the bulk structure [46]. This change revealed the transfer of electrons from metallic Ag levels to ZnO nanoparticles, producing of Ag with a unit valence. The interaction of Ag and ZnO nanoparticles was also presented to control the position of the Fermi level of Ag and ZnO NPs, potentially leading to the establishment of a new Fermi level for metallic Ag. Because the conduction band of ZnO was vacant, free electrons above the new Fermi level may be tunneled to it [48]. The C1s peak in the composite structure is observed at 284.78 eV, where Zn–C bonding is responsible for that peak, which shows Carbon binding to Zn in the ZnO lattice [13]. The main binding energy is assigned to the C–C bonding. The binding energy of another peak is accredited to the C=O bonding. It is shown that the peak intensity of C=O bonding for ZnO–Ag-Gp in Fig. 4(d) is higher than that of undoped ZnO in Fig. 4(d). Notably, ZnO–Ag-Gp improved the adsorption quality of ZnO, which can enhance its photocatalytic activity and stability [23]. The atomic% of the elements is listed in Table S3. ZnO with more oxygen vacancies exhibited more photocatalytic activity [49].

The investigation of the thermal stability of prepared composites could be explained by thermogravimetric analysis (TGA). All samples are stable as shown in Figure S7 from a temperature range of 50°C-450 °C. The carbon was decomposed at 450 °C [50].

### 3.3 Optical properties of the prepared samples

**Fig. 5:**
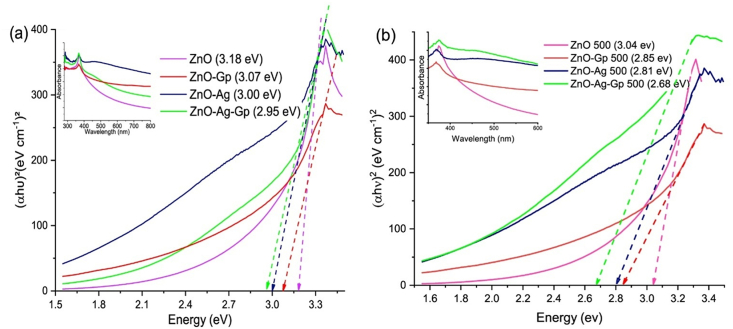
Optical absorbance and Bandgap of Prepared ZnO, ZnO-Gp, ZnO–Ag, ZnO–Ag-Gp catalysts a) Before annealing, b) After annealing.

UV–vis absorption spectrum of photocatalyst is shown in the inset of Fig. 5(a) and (b). The wavelength and absorbance graph was measured by using the solution. This graph indicated a sharp absorption edge close to the UV region at about 368 nm, which showed the intrinsic band-gap of the absorption of the catalysts [51]. A redshift of the absorption peak was identified for ZnO–Ag composites, similar to Kumar et al. [20]. The sharp peaks identified the nanoparticle and narrow size distribution [51]. The prepared nanocomposites' optical band gap (Eg) were evaluated from the corresponding Tauc's plots as shown in Fig. 5(b) bandgap of catalysts before and after annealing following equation by Kubelka–Munk model [52].

(12) $${\left(\alpha h\nu \right)}^{n}=C\left(h\nu -E_{g}\right)$$

Here n = 2 was used in Eq. (12), giving the direct band-gap with the effective linear fit on a generalization of the Tauc Plot in Fig. 5(a and b) [53].

The bandgap (E_g_) of the prepared pure ZnO photocatalyst was 3.18 eV and decreased to 2.95 eV for the ZnO–Ag-Gp photocatalyst. This result was nearly the same as the investigation of A.G.Abd-Elrahim et al. [53]. They investigated that the optical bandgap was reduced of nanostructured ZnO with graphene species incorporation, where graphene nanosheets enhanced the photodegradation property of nanostructured ZnO thin film under solar radiation [53]. The investigation of the bandgap was 3.13eV for ZnO by Kumar et al. and this value decreased with doping of Ag [13]. This study also revealed the bandgap of pure ZnO NPs reduced from 3.18 eV to 3.00 eV for ZnO–Ag in Table 3. That was due to Ag's inclusion, which causes intermediate states to form in the middle of the conduction and valence bands of the pure material host matrix, causing the bandgap of ZnO–Ag to reduce. Silver acts as an acceptor material, causing the bandgap of ZnO NPs to alter and so decrease. In contrast, the donor materials cause a blue shift and increase the bandgap in ZnO nanostructures. The bandgap of catalysts decreased after annealing temperature [52].

**Table 3:** Comparative study of the bandgap before and after annealing samples.

Photocatalysts	Bandgap before annealing (eV)	Bandgap after annealing at 500 °C (eV)
ZnO	3.18	3.04 eV
ZnO-Gp	3.07	2.85 eV
ZnO–Ag	3.00	2.81 eV
ZnO–Ag-Gp	2.95	2.65 eV

### 3.4 Photodegradation of CIP

**Fig. 6:**
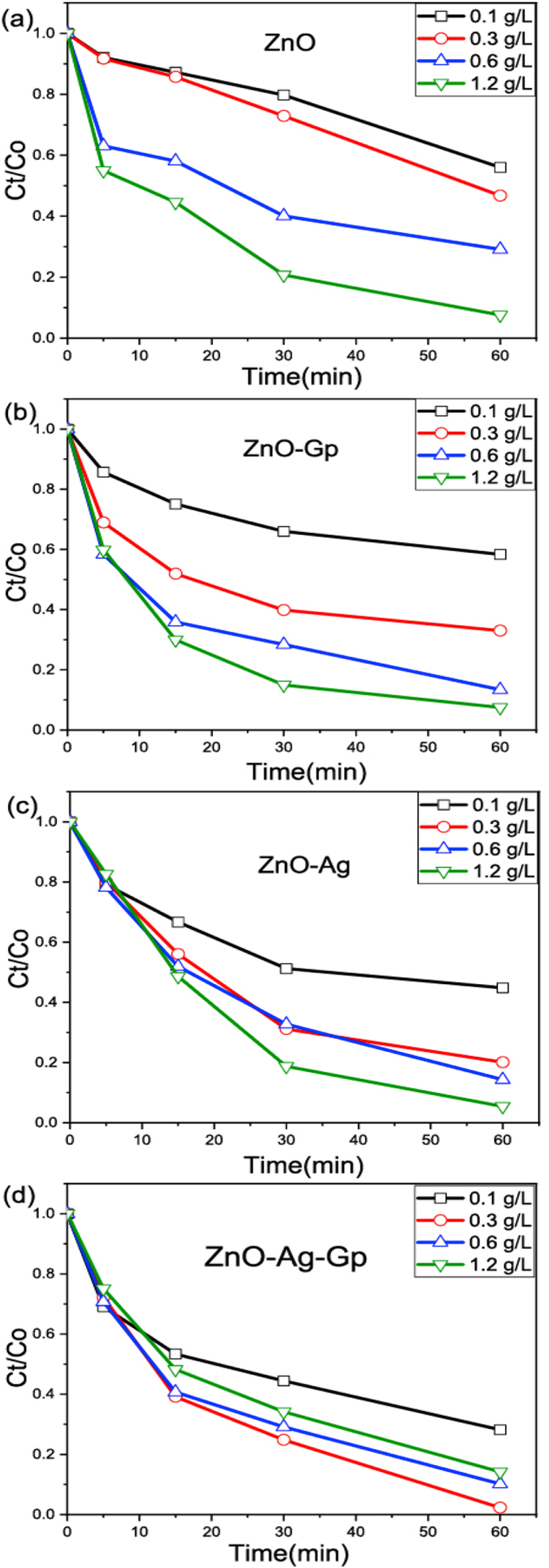
Photodegradation of CIP with different dosage (0.1 g/L, 0.3 g/L,0.6 g/L and 1.2 g/L) of different catalysts (a) ZnO, (b) ZnO-Gp, (c) ZnO–Ag, (d) ZnO–Ag-Gp. Supplementary information provided changes in the absorbance spectra of CIP with (a) ZnO and (b) ZnO–Ag-Gp photocatalyst (Figure S8 in Supplementary Information).

CIP degradation by prepared catalysts was studied under UV short wavelength (254 nm) irradiation. The parameters optimization such as catalyst dosages and different catalysts were investigated to achieve the best conditions for efficient photocatalytic degradation. The efficiency of catalyst doses (0.1–1.2 g/L) with ZnO, ZnO–Ag, ZnO-Gp, and ZnO–Ag-Gp on CIP degradation is shown in Fig. 6 (a, b, c, d). It also revealed that the degradation rate is higher for high doses for all combinations. Only for ZnO–Ag-Gp, there was an optimum dose (0.3 g/L) above which efficiency decreases. These results showed that Fig. 6 ZnO, ZnO–Ag, and ZnO-Gp had the highest efficiency (1.2 g/L) because the degradation rate increased with the increasing catalyst dose [54]. The optimum dose concentration of ZnO–Ag-Gp was 0.3 g/L. Because as the catalyst dose was increased, more active sites were created, resulting in a faster degradation rate [54]. However, if the catalyst's doses were increased further, the UV radiation could be blocked by the dissipated particles of the photocatalyst, reducing the degradation efficiency [11,33]. Furthermore, agglomeration and coagulation might occur at high catalyst dosages, reducing the surface area accessible for photodegradation activation [11].

**Fig. 7:**
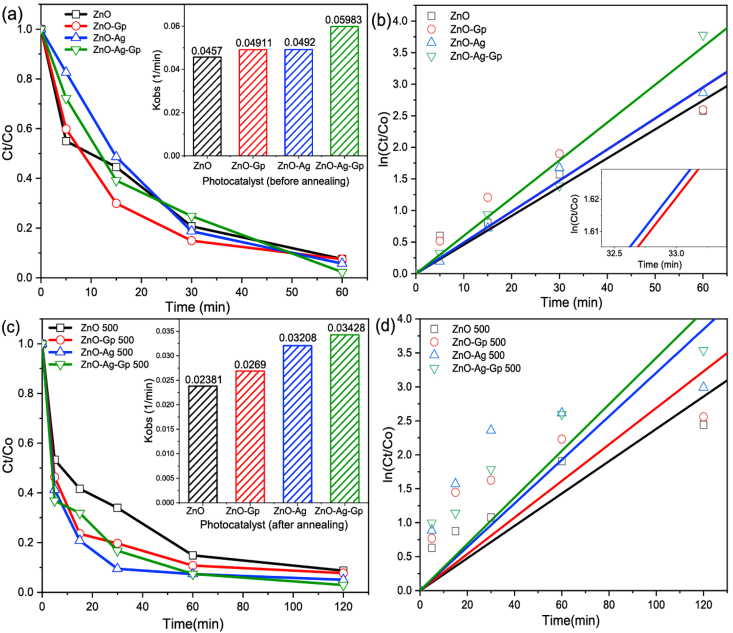
Photocatalytic degradation of CIP (a) and (b) before annealing over 60 min (c) and (d) after annealing over 120 min samples at maximum load condition (ZnO, ZnO–Ag, ZnO-Gp with 1.2 g/L and optimum load of ZnO–Ag-Gp with 0.3 g/L).

The degradation of CIP exhibited in Fig. 7(a, b, c, d) was studied for different catalysts, such as ZnO, ZnO–Ag, ZnO-Gp for their highest catalyst dose and ZnO–Ag-Gp at their optimum dose concentration. ZnO–Ag-Gp in Fig. 7(a and b) exhibited more efficiency than other catalysts. The degradation of concentrations with time of the UV–vis spectrum with the influence of ZnO–Ag-Gp and ZnO–Ag-Gp 500 photocatalysts under 60 min and 120 min is observed in Fig. 7 respectively. The photodegradation of CIP by prepared ZnO was about 92% after 60 min. The efficiency over ZnO-Gp was slightly increased from 92% to 93% after 60 min of UV irradiation. ZnO–Ag modified the degradation of CIP from 92% to 94% after 60 min in Fig. 7(a, b). So, the effect of Ag/Gp individually was not significant. The efficiency of ZnO–Ag-Gp increased from 92% to 98%. The doping effect of Ag in modifying the local and electronic structure improved the light energy for ZnO, according to Samadi et al. [55]. Carbon sheets and Ag metal act as electron sinks, considerably lowering photoinduced electron and hole recombination [11]. So, this study revealed that when Gp/Ag was added, a modified effect was found. Graphite has adsorbance property [23,42] and photocatalytic phenomena [11].

A few prior reports on photocatalytic degradation of CIP using ZnO and other materials doped ZnO samples are summarized in Table 4. Among them, Gupta et al. [56] worked on the synthesis of polymeric S–C3N4/ZnO-Chitosan (SCZ-CH) hydrogels to remove 93% Ciprofloxacin under UV light. They analyzed the satisfactory photocatalytic quality of SCZCH hydrogels performed due to their multifaceted characteristics. Du et al. [25] investigated 87.1% removal of CIP within 120 min under visible light using ZnO/Ag/Ag_3_PO_4_ composite prepared by simple precipitation deposition method and photoreduction technology. They discovered that Ag nanoparticles can operate as a charge transferring bridge between Ag_3_PO_4_ and ZnO, allowing photogenerated electrons to be transferred and separated more quickly. Song et al. [26] reported production of Z-scheme multi-shelled ZnO/AgVO_3_ composite prepared by calcination of carbon sphere template and in situ growth method and investigated 97.12% degradation of CIP over 120 min. They suggested the carbon templates were involved in this experiment and high calcination (400–500°c) temperature was used.

**Table 4:** An overview of the ability of degradation (%) of CIP (Pseudo 1st order) of ZnO and doped ZnO catalysts.

Photocatalysts	Dosage g/L	CIP with concentration mg/L	Light source	Degradation time(min)	Percentage of degradation(%)	Ref. year
ZnO	0.2	5	UV	60	48	[57] 2010
N–ZnO/CdS/GO	0.3	15	300 W Xenon lamp	60	86	[27] 2016
ZnO/Ag2O	0.8	10	250 W UV lamp	60	31	[58] 2017
Fe Doped ZnO	0.15	10	Sunlight intensity of 120,000–135,000 lux	210	66	[10] 2018
Gd2WO6/ZnO/bentonite	0.5	20	Tungsten lamp (150 mW/cm^2^)λ > 400 nm	60	97.9	[33] 2019
ZnO/AgVO3	0.1	10	300 W Xe lamp	120	97.12	[26] 2020
ZnO/Ag/Ag3PO4	0.5	10	300 W xenon lamp λ < 420 nm	120	87.1	[25] 2021
S–C3N4/ZnO-Chitosan	2	20	UV-A tube light (30 W)	300	93	[56] 2022
ZnO	1.2	5	24 W UV lamp λ = 254 nm	60	92	Present study
ZnO-Gp	1.2	5	24 W UV lamp λ = 254 nm	60	93	Present study
ZnO–Ag	1.2	5	24 W UV lamp λ = 254 nm	60	94	Present study
ZnO–Ag-Gp	0.3	5	24 W UV lamp λ = 254 nm	60	98	Present study

However, according to our knowledge, the degradation of CIP with ZnO–Ag-Gp composite in the current study is entirely new. Interestingly most of the earlier studies indicated that the photodegradation of CIP (pharmaceutical pollutants) utilizing ZnO–Ag-Gp is still the most efficient. Degradation of CIP over ZnO–Ag-Gp exhibited 98% after 60 min under UV irradiation without any external agent. No temperature treatment is essential for this production and degradation of pharmaceutical pollutants. Moreover, this study used a Pseudo 1st order equation to determine degradation efficiency and the acceptance of 1st order compared to the 0th and 2nd order as given in Supplementary Information (Figure S9).

From Table 5, it is observed that in this present study, at an annealing temperature of 500 °C, all the prepared catalysts showed a slightly decreasing value of degradation efficiency and significantly decreasing the rate constants (1/min) compared with the prepared catalysts before annealing. Because photocatalytic reactions occur mainly on the catalyst surface, the photodegradation of a semiconductor is intimately connected to its internal particle size, shape, and surface feature i.e. synthesis conditions [33,59]. Due to the deterioration of the textural qualities, the degradation efficiency decreases as the annealing temperature rises [60].

**Table 5:** Comparative study of the Rate constants (1/min) before and after annealing.

Photocatalysts	Degradation time(min)		% of degradation		Kinetics Model	Rate constant (1/min)	
	before annealing	after annealing	before annealing	after annealing		before annealing	after annealing
ZnO	60	120	92	91.3	Pseudo 1st Order	0.0457	0.02381
ZnO-Gp	60	120	93	92.2		0.04911	0.0269
ZnO–Ag	60	120	94	94.9		0.0492	0.03208
ZnO–Ag-Gp	60	120	98	97.5		0.05983	0.03428

The rate of CIP degradation (k_obs_) increased from 0.03428 min^−1^ by sample (after annealing) to 0.05983 min^−1^ by sample (before annealing) ZnO–Ag-Gp as shown in Table 5**.** CIP degradation over ZnO–Ag-Gp (before annealed) and ZnO–Ag-Gp 500 (after annealed) became 98% and 97.5% after 60 min and 120 min, respectively. It is noticeable that the simplicity of the mechanism for high efficiency, there is no need to annealing at a higher temperature of that composite. Because of environmental issues, removing the pharmaceuticals pollutants from wastewater at a very large scale should easily develop.

### 3.5 Photocatalytic stability and mechanism

**Fig. 8:**
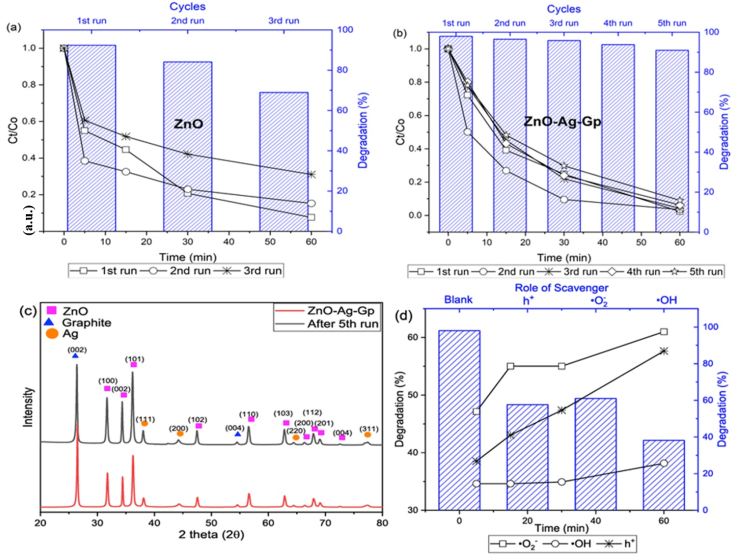
Photodegradation stability of (a) ZnO: catalyst dose 1.2 g/L and (b) ZnO–Ag-Gp: catalyst dose 0.3 g/L (c) XRD of ZnO–Ag-Gp samples before and after completing 5 cycles of photocatalysis and (d) Scavenging study to investigate the driving radical for the CIP degradation through ZnO–Ag-Gp (0.3 g/L) (Conditions: concentration of CIP 5 mg/L time:60 min).

To assess the photostability of CIP, five recycle tests were taken, which are shown in Fig. 8(a, b, c, d). CIP removal was 98% for ZnO–Ag-Gp after 60 min of irradiation for the first time. The efficiency did not vary considerably in the second run, although it did drop from 98% to 96.49% from the first. These experiments demonstrated that the photocatalyst is highly stable even after five cycles (90.97%), however, small reductions in degradation were found, having material loss during the cleaning and centrifugation operations. At the same time, the efficiency of ZnO was reduced to 68.93% after 3rd run. The photostability of ZnO was not sufficient. Notably, Ag doping and hybridization with graphite improve the photocatalytic activity while also improving their stability. By the XRD pattern of ZnO–Ag-Gp in Fig. 8, there was no change even after 5 cycles of reusability of photodegradation of CIP. The results established was relatable with the work of the use of produced Silver doped ZnO hyridizing Graphite that showed the removal reached to 75.8% from 88.5% [11].

The main trapping radicals such as the •OH, h^+^ and •O_2_^−^ are identified through scavenging study using scavengers such as Isopropyl Alcohol (IPA), Ethylene-diaminetetra-acetic acid disodium salt dihydrate (EDTA-2Na), and Acrylamide, respectively. The presence of •OH, h^+^, and •O_2_^−^ scavengers reduced the CIP degradation efficiency to 38.16%, 57.65%, and 61%, respectively, in Fig. 8, whereas without scavengers, it was 98%. This result investigated that •OH radical acted as the main trapping radical, whereas h^+^ and •O_2_^−^ acted as secondary radicals for CIP degradation. This result was close to the earlier report by Sharmin and Basith [45] and Mohamed et al. [61]. The decomposition products were low molecular organic acids and Fluride ion released from F^−^ group of CIP molecules reported by Sayed et al. in their work with CIP through AOP method [62] As confirmed from XPS analysis, a peak was activated in the hydroxyl group due to structural defects. It might play a prominent part in the photocatalytic activity because they protect from the rejoining of electron hole pairs. Degradation by-products might be transformed into H_2_O and CO_2_ for CIP mineralization [2].

## 4 Conclusions

This research presented an easily scalable and low temperature hydrothermal method to prepare the composites to show their ability in removing Ciprofloxacin from an aqueous medium. The work reported excellent crystallinity of the nanocomposites that was rendered by XRD and SAED techniques. TEM and FESEM strongly revealed the nanorod ZnO with nanoparticle Ag randomly distributed on a Gp sheet. The Gp sheets overlapped and created a structure of a three-dimensional network of ZnO nanorods was significant because it gave a fast-conducting channel for the electrons. The photodegradation quality was illuminated by their multifaceted properties, including the reduced bandgap energy and highest aspect ratio of ZnO nanorod for the ZnO–Ag-Gp sample compared to the other samples. The most efficient ZnO–Ag-Gp catalyst exhibited 98% degradation of CIP at 60 min and 97.5% by annealed samples (ZnO–Ag-Gp 500) after 120 min. The sample (before annealing) ZnO–Ag-Gp yielded the most considerable k_obs_ value, which was 1.75 times larger than the sample (after annealing) ZnO–Ag-Gp 500. The XPS study disclosed that the hydroxyl group activated the peak due to structural defects. These groups might play a prominent part in photocatalytic activity because they protect from the rejoining of electron-hole pairs. By the help of the scavenger experiment, it has been showed that the •OH radicals played the primary role in the degradation of CIP. The TGA recorded the decomposition of carbon started at 450 °C. So, it is redundant to apply annealed composites and that resumption concludes our time-saving and unembellished process. The findings of this research might suggest new perspectives on an industrial scale and the above method is expected to be cost-effective for the degradation of antibiotics in wastewater treatment for environmental purposes.

### Author contribution statement

Tanu Shree Roy, Md. Abdul Gafur: Conceived and designed the experiments; Performed the experiments; Analyzed and interpreted the data; Contributed reagents, materials, analysis tools or data; Wrote the paper.

Surya Akter: Conceived and designed the experiments; Analyzed and interpreted the data; Contributed reagents, materials, analysis tools or data; Wrote the paper.

Monabbir Rafsan Fahim: Analyzed and interpreted the data; Wrote the paper.

Tahmina Ferdous: Conceived and designed the experiments; Performed the experiments; Analyzed and interpreted the data; Contributed reagents, materials, analysis tools or data; Wrote the paper.

## Funding statement

This research did not receive any specific grant from funding agencies in the public, commercial, or not-for-profit sectors.

## Data availability statement

Data will be made available on request.

## Declaration of interest's statement

The authors declare no competing interests.

## References

[1] Liu X., Steele J.C., Meng X.Z. (2017). Usage, residue, and human health risk of antibiotics in Chinese aquaculture: a review. Environ. Pollut..

[2] Hassani A., Khataee A., Karaca S., Karaca C., Gholami P. (2017). Sonocatalytic degradation of ciprofloxacin using synthesized TiO2 nanoparticles on montmorillonite. Ultrason. Sonochem..

[3] Karuppaiah S. (2019). Efficient photocatalytic degradation of ciprofloxacin and bisphenol A under visible light using Gd 2 WO 6 loaded ZnO/bentonite nanocomposite. Appl. Surf. Sci..

[4] Jiang J.Q., Zhou Z., Pahl O. (2012). Preliminary study of ciprofloxacin (cip) removal by potassium ferrate(VI). Separ. Purif. Technol..

[5] Kaur A., Anderson W.A., Tanvir S., Kansal S.K. (2019). Solar light active silver/iron oxide/zinc oxide heterostructure for photodegradation of ciprofloxacin, transformation products and antibacterial activity. J. Colloid Interface Sci..

[6] Redgrave L.S., Sutton S.B., Webber M.A., Piddock L.J.V. (2014). Fluoroquinolone resistance: mechanisms, impact on bacteria, and role in evolutionary success. Trends Microbiol..

[7] Cheng S., Mao Z., Sun Y., Yang J., Yu Z., Gu R. (2021). A novel electrochemical oxidation-methanogenesis system for simultaneously degrading antibiotics and reducing CO2 to CH4 with low energy costs. Sci. Total Environ..

[8] Goncalves M.G., da Silva Veiga P.A., Fornari M.R., Peralta-Zamora P., Mangrich A.S., Silvestri S. (2020). Relationship of the physicochemical properties of novel ZnO/biochar composites to their efficiencies in the degradation of sulfamethoxazole and methyl orange. Sci. Total Environ..

[9] Elmolla E.S., Chaudhuri M. (2010). Comparison of different advanced oxidation processes for treatment of antibiotic aqueous solution. Desalination.

[10] Das S. (2018). Sunlight assisted photocatalytic degradation of ciprofloxacin in water using fe doped zno nanoparticles for potential public health applications. Int. J. Environ. Res. Publ. Health.

[11] Tran M.L., Nguyen C.H., Fu C.C., Juang R.S. (2019). Hybridizing Ag-Doped ZnO nanoparticles with graphite as potential photocatalysts for enhanced removal of metronidazole antibiotic from water. J. Environ. Manag..

[12] Fu S. (2019). Few-layer WS2 modified BiOBr nanosheets with enhanced broad-spectrum photocatalytic activity towards various pollutants removal. Sci. Total Environ..

[13] Kumar S., Singh V., Tanwar A. (2016). Structural, morphological, optical and photocatalytic properties of Ag-doped ZnO nanoparticles. J. Mater. Sci. Mater. Electron..

[14] Ceretta M.B., Vieira Y., Wolski E.A., Foletto E.L., Silvestri S. (2020). Biological degradation coupled to photocatalysis by ZnO/polypyrrole composite for the treatment of real textile wastewater. J. Water Proc. Eng..

[15] Klavarioti M., Mantzavinos D., Kassinos D. (2009). Removal of residual pharmaceuticals from aqueous systems by advanced oxidation processes. Environ. Int..

[16] Raj R.B., Umadevi M., Parimaladevi R. (2021). Effect of ZnO/Ag nanocomposites against anionic and cationic dyes as photocatalysts and antibacterial agents. J. Inorg. Organomet. Polym. Mater..

[17] Shah A.H., Basheer Ahamed M., Manikandan E., Chandramohan R., Iydroose M. (2013). Magnetic, optical and structural studies on Ag doped ZnO nanoparticles. J. Mater. Sci. Mater. Electron..

[18] Qi K., Cheng B., Yu J., Ho W. (2017). Review on the improvement of the photocatalytic and antibacterial activities of ZnO. J. Alloys Compd..

[19] Jun M.C., Park S.U., Koh J.H. (2012). Comparative studies of Al-doped ZnO and Gadoped ZnO transparent conducting oxide thin films. Nanoscale Res. Lett..

[20] Singhal S., Kaur J., Namgyal T., Sharma R. (2012). Cu-doped ZnO nanoparticles: synthesis, structural and electrical properties. Phys. B Condens. Matter.

[21] Kim K., Lee D.H., Lee S.Y., Jang G.E., Kim J.S. (2012). Effect of Ag/Al co-doping method on optically p-type ZnO nanowires synthesized by hot-walled pulsed laser deposition. Nanoscale Res. Lett..

[22] Peng Y., Ji J., Chen D. (2015). Ultrasound assisted synthesis of ZnO/reduced graphene oxide composites with enhanced photocatalytic activity and anti-photocorrosion. Appl. Surf. Sci..

[23] Chen T. (2016). Enhanced photocatalytic activity of C@ZnO core-shell nanostructures and its photoluminescence property. Appl. Surf. Sci..

[24] Umukoro E.H. (2017). Photocatalytic application of Pd-ZnO-exfoliated graphite nanocomposite for the enhanced removal of acid orange 7 dye in water. Solid State Sci..

[25] Du C. (2021). Facile synthesis of Z-scheme ZnO/Ag/Ag3PO4 composite photocatalysts with enhanced performance for the degradation of ciprofloxacin. Mater. Chem. Phys..

[26] Song S., Wu K., Wu H., Guo J., Zhang L. (2020). Synthesis of Z-scheme multi-shelled ZnO/AgVO3 spheres as photocatalysts for the degradation of ciprofloxacin and reduction of chromium(VI). J. Mater. Sci..

[27] Huo P. (2016). Incorporation of N–ZnO/CdS/Graphene oxide composite photocatalyst for enhanced photocatalytic activity under visible light. J. Alloys Compd..

[28] Wei H., Wu Y., Lun N., Hu C. (Feb. 2005). Hydrothermal synthesis and characterization of ZnO nanorods. Mater. Sci. Eng..

[29] Ha T.T., Canh T.D., Tuyen N.V. (2013). A quick process for synthesis of ZnO nanoparticles with the aid of microwave irradiation. ISRN Nanotechnology.

[30] Sagadevan S., Pal K., Chowdhury Z.Z., Hoque M.E. (2017). Structural, dielectric and optical investigation of chemically synthesized Ag-doped ZnO nanoparticles composites. J. Sol. Gel Sci. Technol..

[31] Hao N.T., Nguyen H., Nguyen L., Do K.N., Vu L.D. (2020). Efficient removal of ciprofloxacin in aqueous solutions by zero-valent metal-activated persulfate oxidation: a comparative study. J. Water Proc. Eng..

[32] Akter S., Islam S., Kabir H., Ali Shaikh A., Gafur A. (2022). UV/TiO2 photodegradation of metronidazole, ciprofloxacin and sulfamethoxazole in aqueous solution: an optimization and kinetic study. Arab. J. Chem..

[33] Salma A., Thoroe-Boveleth S., Schmidt T.C., Tuerk J. (2016). Dependence of transformation product formation on pH during photolytic and photocatalytic degradation of ciprofloxacin. J. Hazard Mater..

[34] Ezeigwe E.R., Tan M.T.T., Khiew P.S., Siong C.W. (2015). One-step green synthesis of graphene/ZnO nanocomposites for electrochemical capacitors. Ceram. Int..

[35] Goswami M., Adhikary N.C., Bhattacharjee S. (2018). Effect of annealing temperatures on the structural and optical properties of zinc oxide nanoparticles prepared by chemical precipitation method. Optik.

[36] Kayani Z.N., Manzoor F., Zafar A., Mahmood M., Rasheed M., Anwar M. (2020). Impact of Ag doping on structural, optical, morphological, optical and photoluminescent properties of ZnO nanoparticles. Opt. Quant. Electron..

[37] Haruna A., Abdulkadir I., Idris S.O. (2020). Effect of annealing temperature on the synthesis and photocatalytic properties of Bi0.65K0.2Ba0.15FeO3 perovskite-like nanoparticle synthesized by sol-gel method. Beni Suef Univ. J. Basic Appl. Sci.

[38] Tian J.L. (2015). Influence of film thickness and annealing temperature on the structural and optical properties of ZnO thin films on Si (1 0 0) substrates grown by atomic layer deposition. Superlattice. Microst..

[39] Thennarasu G., Sivasamy A. (2016). Enhanced visible photocatalytic activity of cotton ball like nano structured Cu doped ZnO for the degradation of organic pollutant. Ecotoxicol. Environ. Saf..

[40] Abdelmohsen A.H., Rouby W.M.A.E., Ismail N., Farghali A.A. (2017). Morphology transition engineering of ZnO nanorods to nanoplatelets grafted Mo8O23-MoO2 by polyoxometalates: mechanism and possible applicability to other oxides. Sci. Rep..

[41] Boulahlib S., Dib K., Özacar M., Bessekhouad Y. (2021). Optical, dielectric, and transport properties of Ag-doped ZnO prepared by Aloe Vera assisted method. Opt. Mater..

[42] Anirudhan T.S., Deepa J.R. (2017). Nano-zinc oxide incorporated graphene oxide/nanocellulose composite for the adsorption and photo catalytic degradation of ciprofloxacin hydrochloride from aqueous solutions. J. Colloid Interface Sci..

[43] Khurshid F., Jeyavelan M., Hudson M.S.L., Nagarajan S. (2019). Ag-doped ZnO nanorods embedded reduced graphene oxide nanocomposite for photo-electrochemical applications. R. Soc. Open Sci..

[44] Wahab R., Ansari S.G., Kim Y.S., Dar M.A., Shin H.S. (2008). Synthesis and characterization of hydrozincite and its conversion into zinc oxide nanoparticles. J. Alloys Compd..

[45] Sharmin F., Basith M.A. (2022). Highly efficient photocatalytic degradation of hazardous industrial and pharmaceutical pollutants using gadolinium doped BiFeO3 nanoparticles. J. Alloys Compd..

[46] Hosseini S.M., Sarsari I.A., Kameli P., Salamati H. (2015). Effect of Ag doping on structural, optical, and photocatalytic properties of ZnO nanoparticles. J. Alloys Compd..

[47] Sahu R.K. (2012). Stabilization of intrinsic defects at high temperatures in ZnO nanoparticles by Ag modification. J. Colloid Interface Sci..

[48] Zheng Y., Zheng L., Zhan Y., Lin X., Zheng Q., Wei K. (2007). Ag/ZnO heterostructure nanocrystals: synthesis, characterization, and photocatalysis. Inorg. Chem..

[49] Tang Y., Zhou H., Zhang K., Ding J., Fan T., Zhang D. (2015). Visible-light-active ZnO via oxygen vacancy manipulation for efficient formaldehyde photodegradation. Chem. Eng. J..

[50] Zhang H., Cen Y., Du Y., S. R.- Sensors, and undefined (2016).

[51] Darroudi M., Sabouri Z., Kazemi Oskuee R., Khorsand Zak A., Kargar H., Hamid M.H.N.A. (2013). Sol-gel synthesis, characterization, and neurotoxicity effect of zinc oxide nanoparticles using gum tragacanth. Ceram. Int..

[52] Zak A.K., Abrishami M.E., Majid W.H.A., Yousefi R., Hosseini S.M. (2011). Effects of annealing temperature on some structural and optical properties of ZnO nanoparticles prepared by a modified sol-gel combustion method. Ceram. Int..

[53] Abd-Elrahim A.G., Chun D.M. (2021). Room-temperature deposition of ZnO-graphene nanocomposite hybrid photocatalysts for improved visible-light-driven degradation of methylene blue. Ceram. Int..

[54] Samadi M., Zirak M., Naseri A., Khorashadizade E., Moshfegh A.Z. (2016). Recent progress on doped ZnO nanostructures for visible-light photocatalysis. Thin Solid Films.

[55] Ahmad K.S., Jaffri S.B. (2018). Phytosynthetic Ag doped ZnO nanoparticles : semiconducting green remediators what Is So Different about Was ist so anders am Neuroenhancement. Open Chem.

[56] Gupta B., Gupta A.K. (2022). Photocatalytic performance of 3D engineered chitosan hydrogels embedded with sulfur-doped C3N4/ZnO nanoparticles for Ciprofloxacin removal: degradation and mechanistic pathways. Int. J. Biol. Macromol..

[57] El-Kemary M., El-Shamy H., El-Mehasseb I. (2010). Photocatalytic degradation of ciprofloxacin drug in water using ZnO nanoparticles. J. Lumin..

[58] Zhao S., Zhang Y., Zhou Y., Zhang C., Fang J., Sheng X. (2017). Ionic liquid-assisted photochemical synthesis of ZnO/Ag 2 O heterostructures with enhanced visible light photocatalytic activity. Appl. Surf. Sci..

[59] Qiu X., Li L., Zheng J., Liu J., Sun X., Li G. (2008). Origin of the enhanced photocatalytic activities of semiconductors: a case study of ZnO doped with Mg2+. J. Phys. Chem. C.

[60] Ivetić T.B. (2014). Effect of annealing temperature on structural and optical properties of Mg-doped ZnO nanoparticles and their photocatalytic efficiency in alprazolam degradation. Ceram. Int..

[61] Mohamed R.M., Ismail A.A., Alhaddad M. (2021). A novel design of porous Cr2O3@ZnO nanocomposites as highly efficient photocatalyst toward degradation of antibiotics: a case study of ciprofloxacin. Separ. Purif. Technol..

[62] Sayed M., Ismail M., Khan S., Tabassum S., Khan H.M. (2016). Degradation of ciprofloxacin in water by advanced oxidation process: kinetics study, influencing parameters and degradation pathways. Environ. Technol..

