# Supplementary material for: Incorporation of Ag-doped ZnO nanorod through Graphite hybridization: Effective approach for degradation of Ciprofloxacin
Source: Heliyon. 2023 Jan 21;9(2):e13130. doi: 10.1016/j.heliyon.2023.e13130 (PMC9950824; doi:10.1016/j.heliyon.2023.e13130)
Supplement: Supplementary_Information [file mmc1.docx]

Supplementary Information

**Incorporation of Ag-doped ZnO nanorod through Graphite hybridization: Effective approach for degradation of Ciprofloxacin**


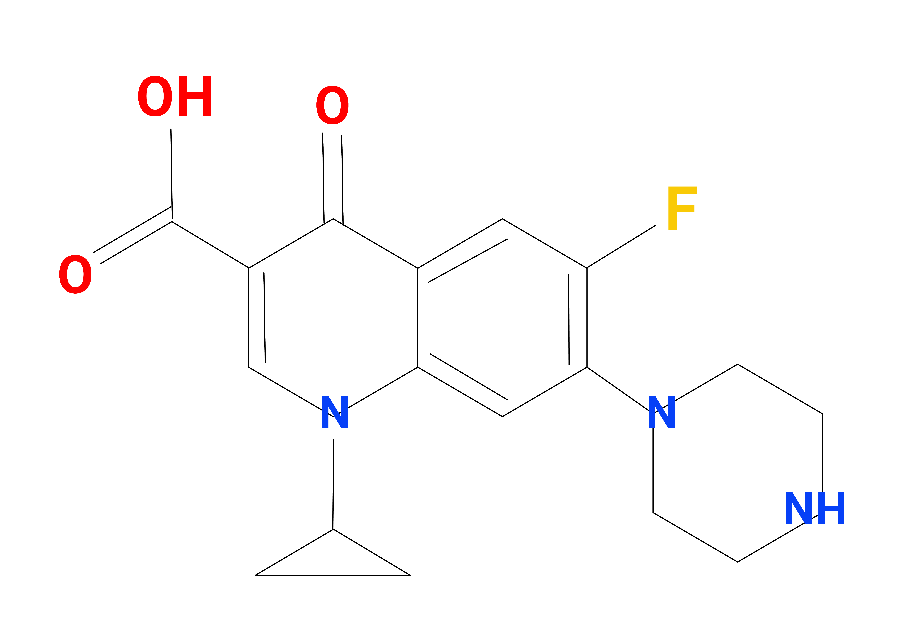


Figure S1: Structure of Ciprofloxacin (C_17_H_18_FN_3_O)


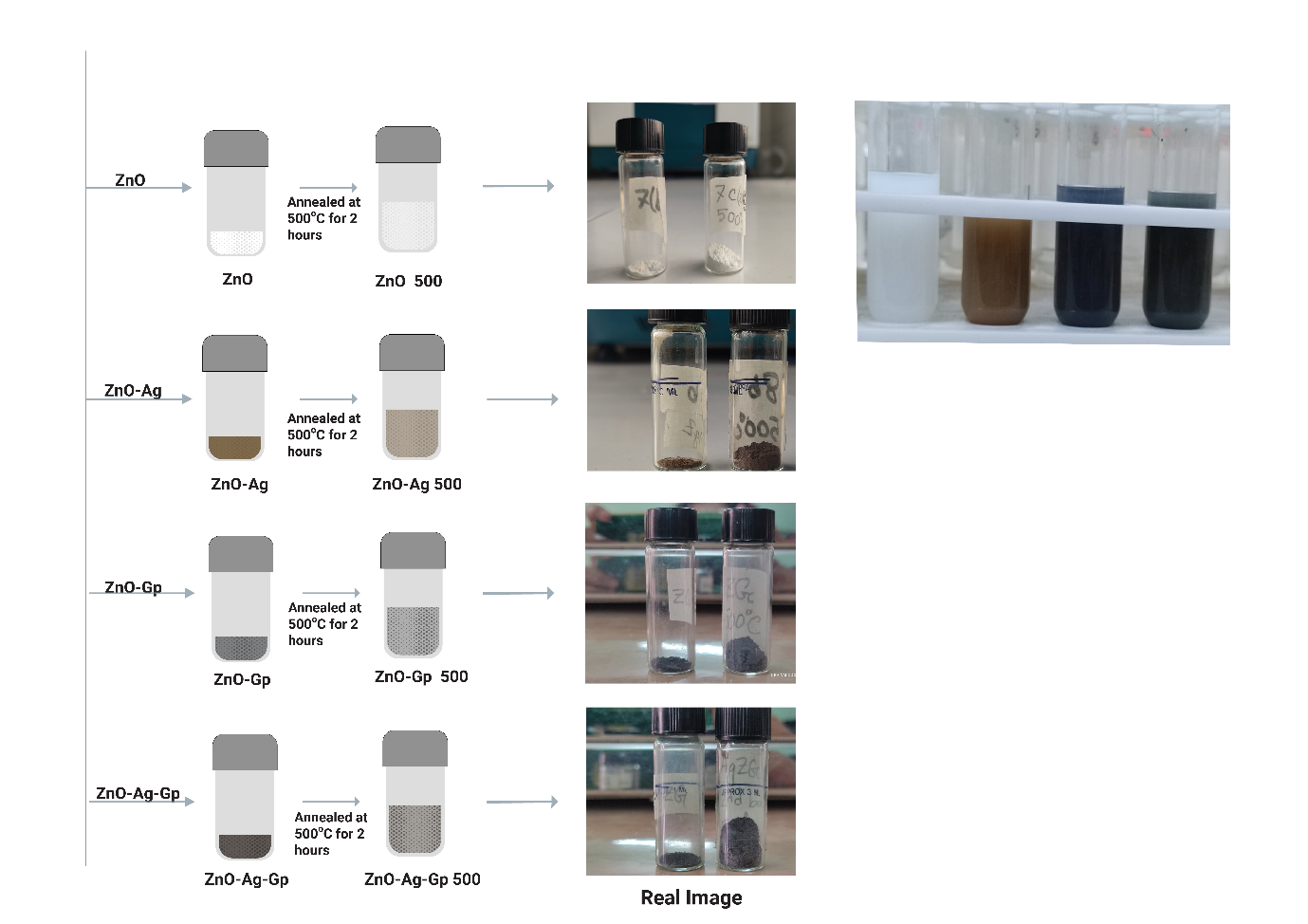


**Figure S2: Produced nanopowder and solution of nanopowder (inset)**

# Fourier Transform Infrared Spectroscopy Analysis

FTIR was used in investigating functional chemical bonding properties of different elements and compounds present in catalysts. Some absorption bands are labelled with a higher wave number, while others are marked with a lower. These bands (peaks) were not all made up of ZnO. These were caused by the occupancy of vapor and carbon dioxide in the environment. Because the pellet was manufactured in a stable environment with dust, water vapors, and CO_2_. The most significant disadvantage of the pressed pellet approach was this. On the other hand, environmental and instrumental factors led to the conclusion that some peaks of water vapor and carbon dioxide were also discovered [1].


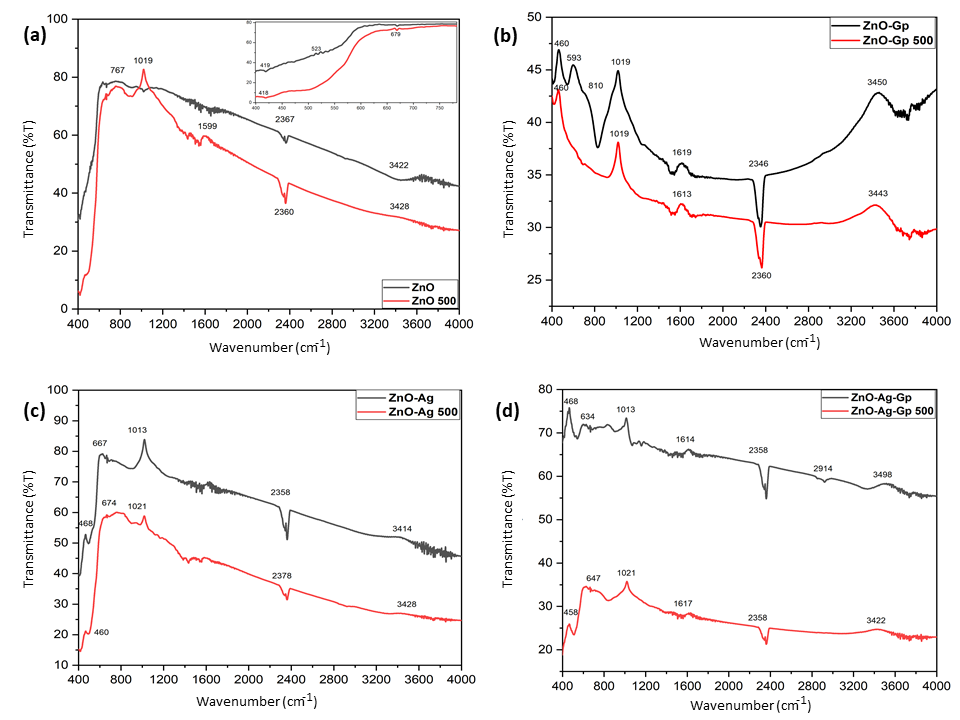


**Figure S3: FTIR analysis of before and after annealing of a) ZnO and ZnO500, b) ZnO-Gp and ZnO-Gp500, c) ZnO-Ag and ZnO-Ag500, d) ZnO-Ag-Gp and ZnO-Ag-Gp500.**

We have observed different peaks in the FTIR spectra of Figure S3, as presented in Table S1, exhibiting the Zn-O, Ag-O, H-O-H,C-O, C=O, and O-H bonding. For the FTIR of prepared ZnO in Figure S3(a), around the peak of 770 cm^-1^ revealed the ZnO existence [1]. The inset <770 cm^-1^ wavenumber identified 419 cm^-1^ and 523 cm^-1^ absorption regions. The wavenumber 1019 cm^-1^and 1599 cm^-1^ indicated the C-O stretching and H-O-H bending, respectively. The absorption peak of 2367 cm^-1^ denoted the presence of CO_2_ molecules in the air for prepared ZnO and the peak of 2360 cm^-1^ for annealed ZnO catalysts[2].

Table S1: Bonding spectra of catalysts

| Infrared absorption bands(cm^-1^) | | | | | | | |
| --- | --- | --- | --- | --- | --- | --- | --- |
| catalysts | Ʋ_1_ (cm^-1^) | Ʋ_2_ (cm^-1^) | Ʋ_3_ (cm^-1^) | Ʋ_4_ (cm^-1^) | Ʋ_5_ (cm-1) | Ʋ_6_ (cm^-1^) | Ʋ_7_ (cm^-1^) |
|  | Zn-O | Ag-O | C-O | H-O-H | C=O | CO_2_ | O-H |
| ZnO | 419 |  | 1019 | 1599 |  | 2367 | 3422 |
| ZnO-Gp | 460 |  | 1018 |  | 1619 | 2346 | 3450 |
| ZnO-Ag |  | 667-850 | 1013 |  |  | 2358 | 3414 |
| ZnO-Ag-Gp | 468 | 634-850 | 1013 |  | 1614 | 2358 | 3498 |

The spectrum of ZnO-Gp prior showed the annealed sample 3443 cm^-1^ in Figure S3(b) which corresponds to the O-H stretching of the surface adsorbed -OH [3].Sharp peaks at 2346 cm^-1^ and 2340 cm^-1^ show the presence of CO_2_ which is present in the air before and after annealing samples, respectively. This result is close to Zhanget. al. investigation[4]. The peak at 460 cm^-1^shows the existence of the ZnO NPs. Vigneshwaran et al. revealed that the FTIR spectra of ZnO showed 480 cm^-1^ due to bonding of Zinc and Oxygen [5]. The wavenumber 419 cm^-1^of the ZnO graph indicates the asymmetric stretching of O–Zn–O associated with the sublattice disorder while the band appears around 523 cm^-1^ may be identified with Oxygen deficiency in ZnO. From the inset of the graph of Figure S3(a), it is observed that the absorption band at 419 cm^-1^and 523 cm^-1^ wavenumbers of ZnO are blue-shifted to 460 cm^-1^and 593 cm^-1^ wavenumbers of the Figure S3(b) graph. The first vibrational alteration shows that the presence of carbon affects the O–Zn–O bond, while the second one might be linked to a decrease in the O/Zn ratio[3]. According to about 500-1000 cm^-1^ absorption region depicted $CO_{3}^{2-}$bonding[4]. The peaks around 1619 cm^-1^ and 1018 cm^-1^ corresponded to the C=O bonding and C-O-C stretching of -COOH groups vibrations respectively[1,3,6]. For Figure S3 (c) of ZnO-Ag sample, the peak at 667 cm^-1^and annealed 674 cm^-1^ absorption regions in the spectra of metal-oxygen stretching modes confirm the incorporation of Ag onto ZnO[7,8]. As a result of Ag incorporating on ZnO, the lengths have increased. When a partial substitution occurred, a transformation occurred cationic silver was incorporated into the ZnO lattice[1]. The peaks at 468 cm^-1^ and 456 cm^-1^(Figure S3 (d)) are almost similar to the peaks around 460 cm^-1^of the sample of ZnO-Gp. The absorption peaks at 634 cm^-1^ and 647 cm^-1^are similar to the ZnO-Ag sample. Therefore, the FTIR analysis may represent that carbon atoms and Ag atoms escorted structural changes in the ZnO structure. The peaks around the wavenumber 1013 cm^-1^, 1021 cm^-1^,1614 cm^-1^, 1619 cm^-1^, 2358 cm^-1^, and 3450 cm^-1^ for annealed sample designated the plane-polarized C-O stretching, C=O bond, presence of CO_2_ molecule due to the air on the surface of the ZnO-Ag-Gp sample, O-H stretching respectively[1,3]. Because the –OH groups can contribute an electron to the photogenerated hole and convert it to reactive OH radicals, the number of –OH groups on the photocatalysts' surfaces are vital to their photocatalytic activity[8,9].The absorption peak at 2914 cm^-1^predicted the C-H stretching mode.

**Figure S4: FESEM and TEM images of (a and b) ZnO-Ag and (c and d) ZnO-Ag-Gp**


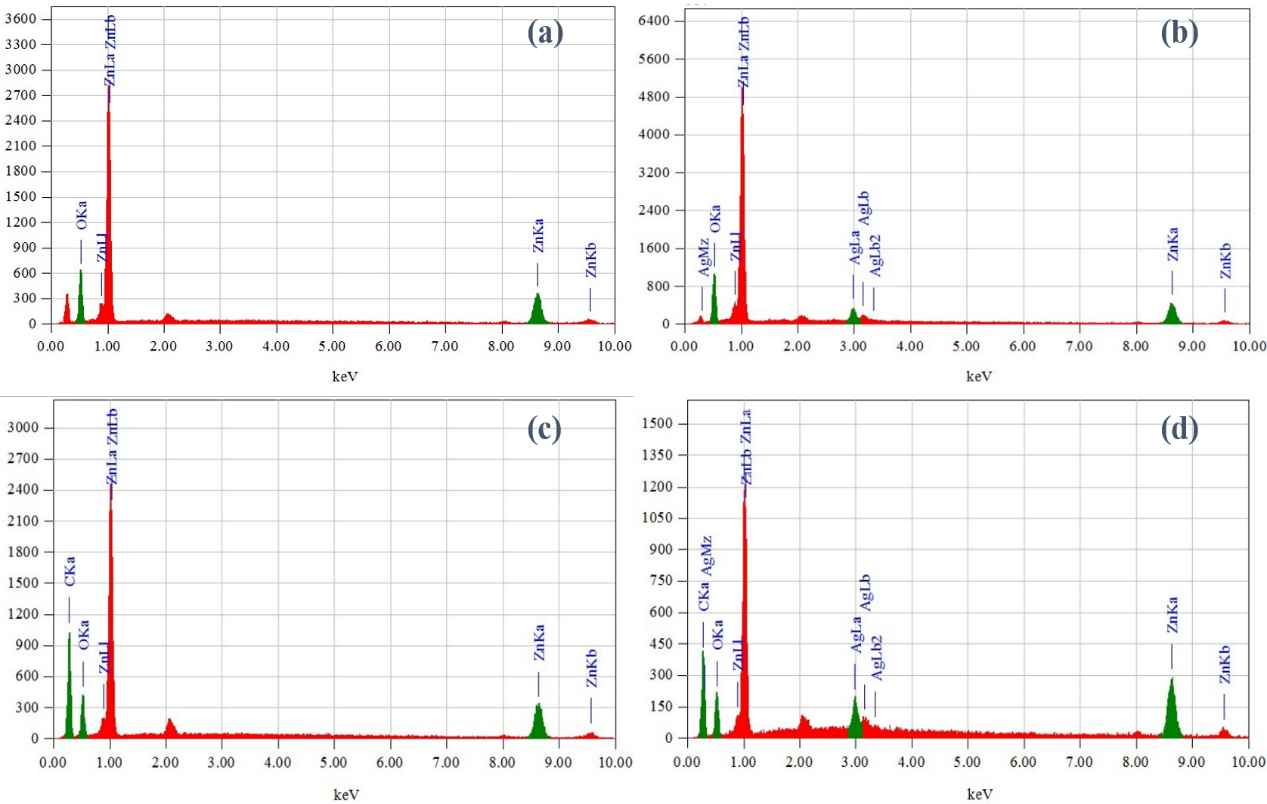


**Figure S5: EDX spectra of (a) ZnO (b) ZnO-Ag (c) ZnO-Gp and (d) ZnO-Ag-Gp**

Table S2: Atomic% and weight% of the traced elements in the prepared samples

| **Materials**  **Elements** | | **ZnO** | **ZnO-Ag** | **ZnO-Gp** | **ZnO-Ag-Gp** |
| --- | --- | --- | --- | --- | --- |
| **Zn** | Weight % | 87.34 | 74.43 | 51.24 | 61.30 |
|  | Atomic % | 62.80 | 50.03 | 16.79 | 25.82 |
| **O** | Weight % | 12.66 | 16.91 | 8.49 | 6.17 |
|  | Atomic % | 37.20 | 46.44 | 11.37 | 10.62 |
| **Ag** | Weight % | -- | 8.66 | -- | 5.41 |
|  | Atomic % | -- | 3.53 | -- | 1.38 |
| **C** | Weight % | -- | -- | 40.27 | 27.12 |
|  | Atomic % | -- | -- | 71.84 | 62.18 |


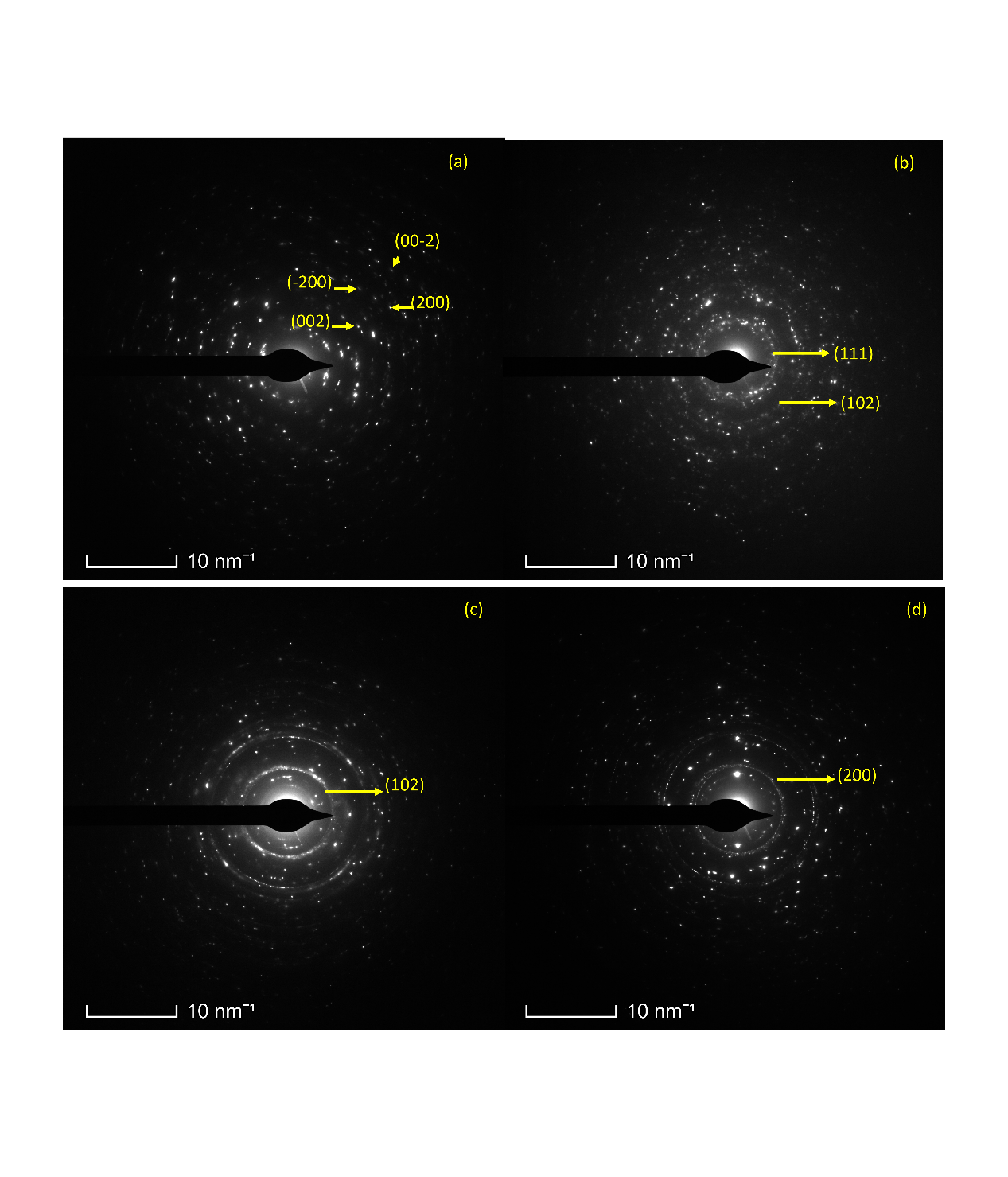


**Figure S6: SAED analysis of (a) ZnO, (b) ZnO-Ag, (c) ZnO-Gp, and (d) ZnO-Ag-Gp samples.**


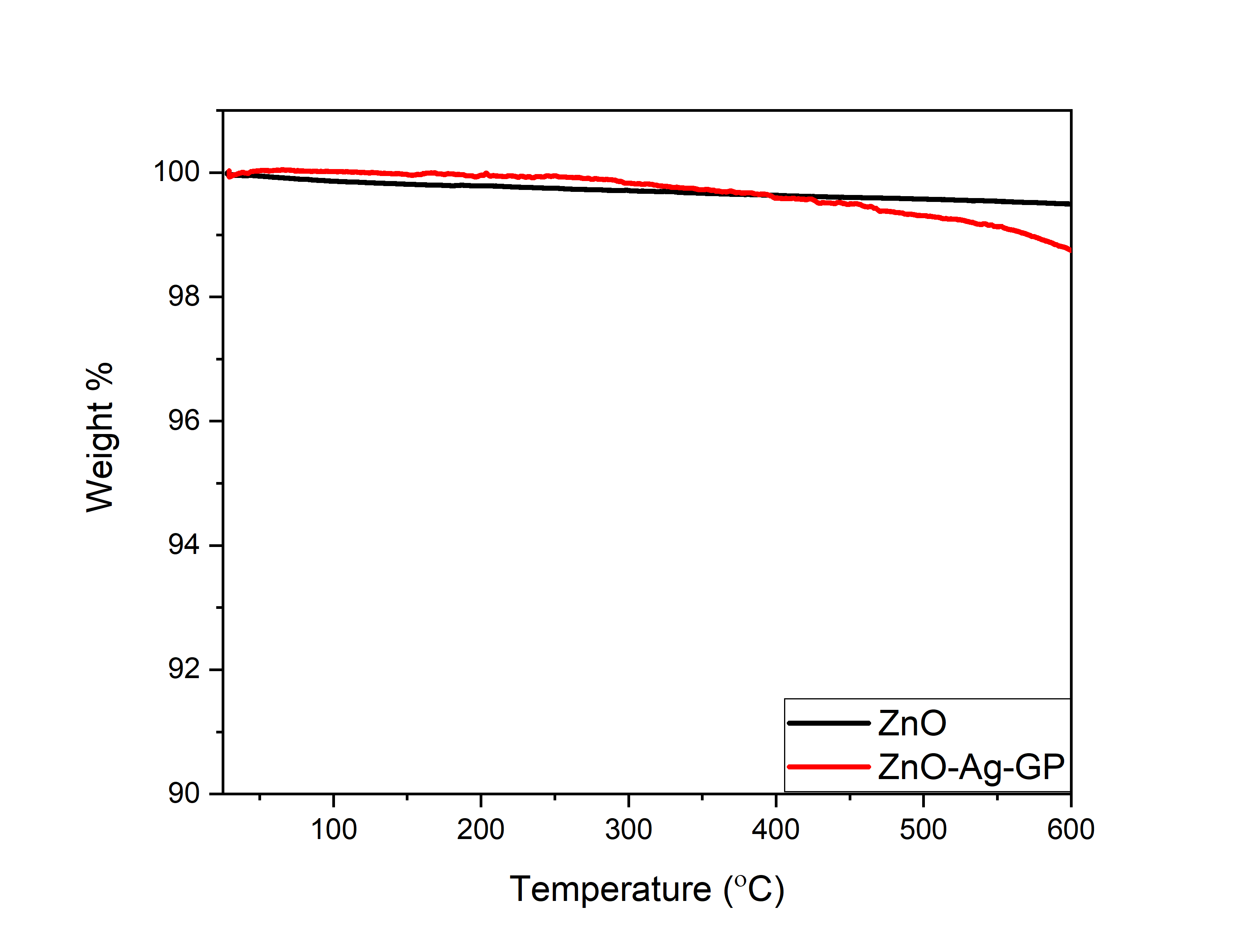


**Figure S7: TGA graph of ZnO and ZnO-Ag-Gp**

**Table S3: Atomic% of the traced elements in the ZnO and ZnO-Ag-Gp sample from XPS**

| **Materials**  **Elements** | | **ZnO** | **ZnO-Ag-Gp** |
| --- | --- | --- | --- |
| **Zn** | Atomic % | 42.04 | 17.5 |
| **O** | Atomic % | 43.47 | 18.47 |
| **C** | Atomic % | 14.49 | 63.59 |
| **Ag** | Atomic % | -- | 0.44 |


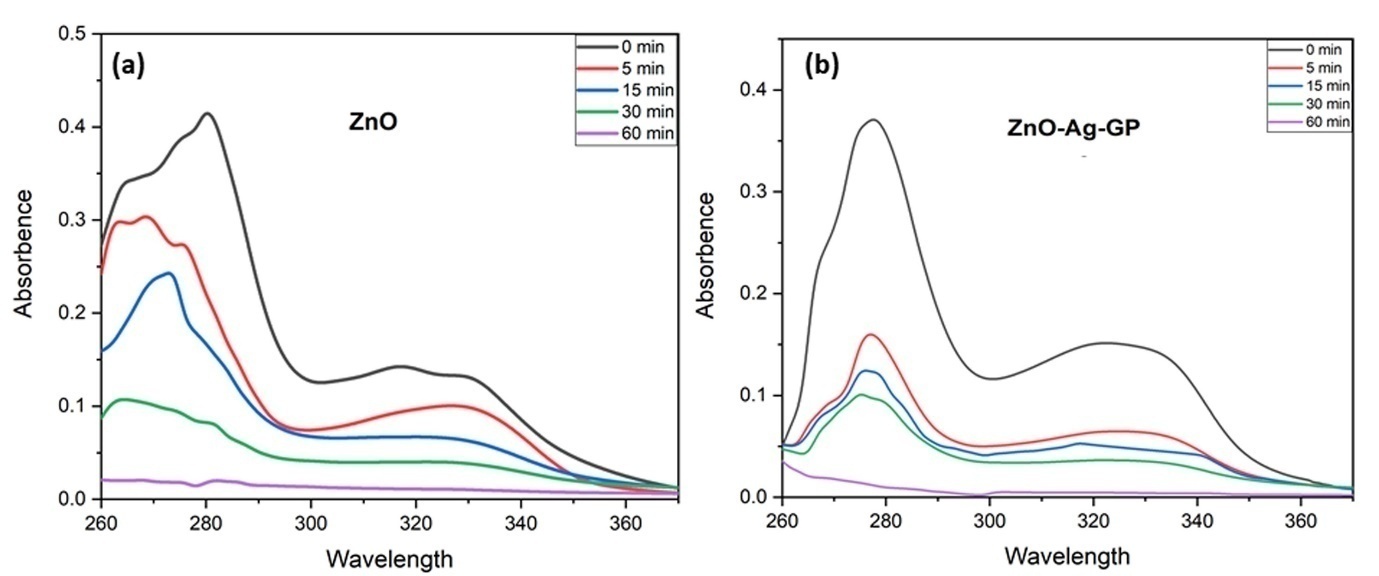


**Figure S8: Changes in the absorbance spectra of CIP with (a) ZnO and (b) ZnO-Ag-Gp photocatalyst**

It is distinguishable that most of the values of R^2^ of 1^st^ order in Figure S9(b) are close to the value of 1, comparatively higher than the others two orders according to Figure S9(a) and Figure S9(c). The graph in Figure S9(d) revealed an appreciable linear fit of 1^st^ order than others. The pseudo1st order kinetics was found to fit concerning 0^th^ and 2^nd^ order kinetics for the degradation of CIP by the AOP method [10,11].


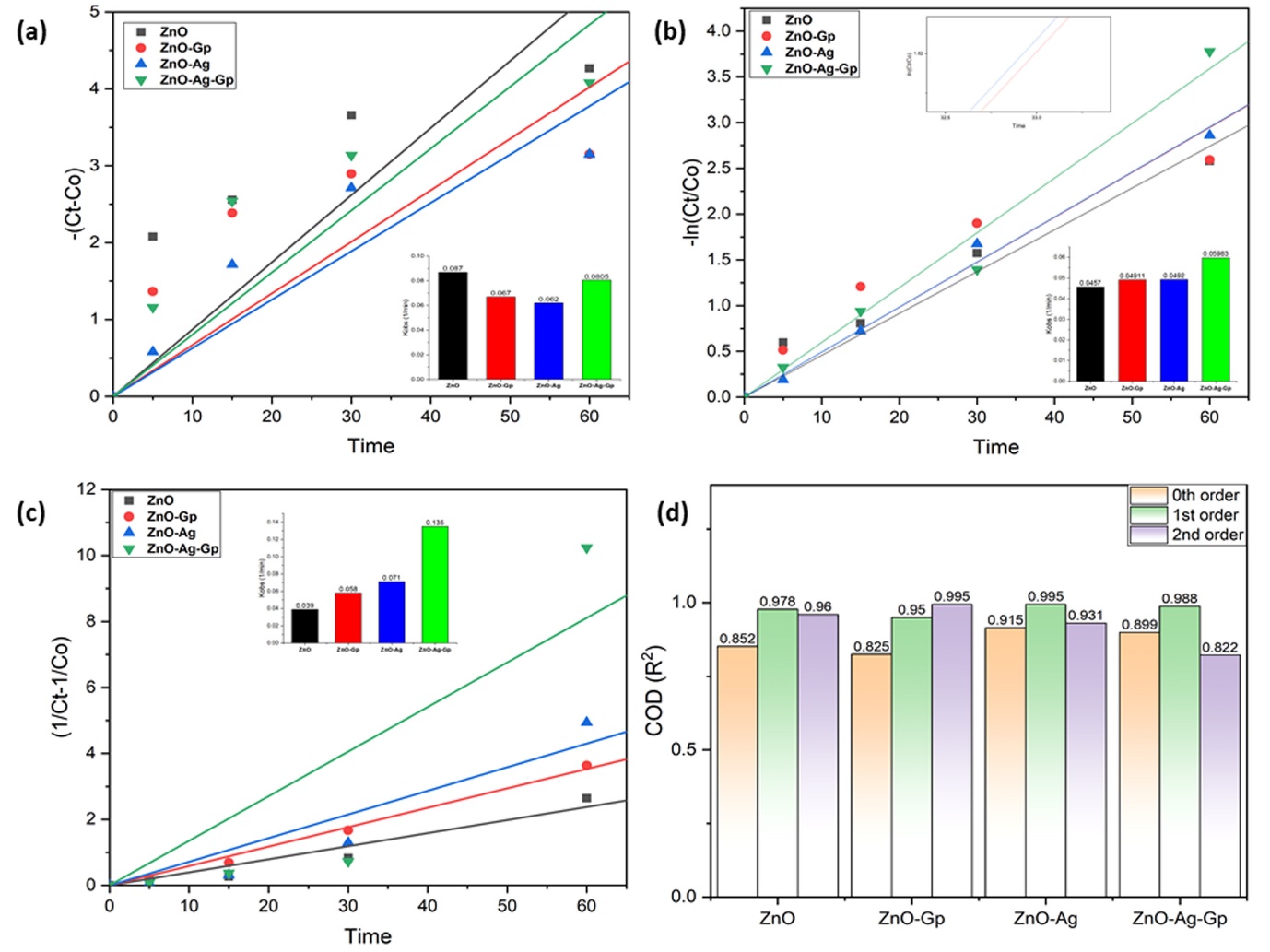


**Figure S9: Equation of degradation of CIP for (a) 0th order, (b)1st order, (c)2nd order, (d)COD value of 0th, 1st, 2nd order of ZnO, ZnO-Gp, ZnO-Ag, and ZnO-Ag-Gp catalysts.**

# References

[1] G. Murtaza, R. Ahmad, M.S. Rashid, M. Hassan, A. Hussnain, M.A. Khan, M. Ehsan Ul Haq, M.A. Shafique, S. Riaz, Structural and magnetic studies on Zr doped ZnO diluted magnetic semiconductor, Curr. Appl. Phys. 14 (2014) 176–181. https://doi.org/10.1016/j.cap.2013.11.002.

[2] L. Wu, Y. Wu, X. Pan, F. Kong, Synthesis of ZnO nanorod and the annealing effect on its photoluminescence property, Opt. Mater. (Amst). 28 (2006) 418–422. https://doi.org/10.1016/j.optmat.2005.03.007.

[3] J.J. Beltrán, C.A. Barrero, A. Punnoose, Relationship between ferromagnetism and formation of complex carbon bonds in carbon doped ZnO powders, Phys. Chem. Chem. Phys. 21 (2019) 8808–8819. https://doi.org/10.1039/c9cp01277j.

[4] Z. Liwu, C. Hanyun, Z. Ruilong, Z. Yongfa, Photocorrosion suppression of ZnO nanoparticles via hybridization with graphite-like carbon and enhanced photocatalytic activity, J. Phys. Chem. C. 113 (2009) 2368–2374. https://doi.org/10.1021/jp807778r.

[5] N. Vigneshwaran, S. Kumar, A.A. Kathe, P. V. Varadarajan, V. Prasad, Functional finishing of cotton fabrics using zinc oxide-soluble starch nanocomposites, Nanotechnology. 17 (2006) 5087–5095. https://doi.org/10.1088/0957-4484/17/20/008.

[6] Y. Peng, J. Ji, D. Chen, Ultrasound assisted synthesis of ZnO/reduced graphene oxide composites with enhanced photocatalytic activity and anti-photocorrosion, Appl. Surf. Sci. 356 (2015) 762–768. https://doi.org/10.1016/j.apsusc.2015.08.070.

[7] K.S. Ahmad, S.B. Jaffri, Phytosynthetic Ag doped ZnO nanoparticles : Semiconducting green remediators What Is So Different About Was ist so anders am Neuroenhancement ?, Open Chem. 16 (2018) 556–570.

[8] Ş.Ş. Türkyılmaz, N. Güy, M. Özacar, Photocatalytic efficiencies of Ni, Mn, Fe and Ag doped ZnO nanostructures synthesized by hydrothermal method: The synergistic/antagonistic effect between ZnO and metals, J. Photochem. Photobiol. A Chem. 341 (2017) 39–50. https://doi.org/10.1016/j.jphotochem.2017.03.027.

[9] K. Kumar, M. Chitkara, I.S. Sandhu, D. Mehta, S. Kumar, Photocatalytic, optical and magnetic properties of Fe-doped ZnO nanoparticles prepared by chemical route, J. Alloys Compd. 588 (2014) 681–689. https://doi.org/10.1016/j.jallcom.2013.11.127.

[10] B. Gupta, A.K. Gupta, Photocatalytic performance of 3D engineered chitosan hydrogels embedded with sulfur-doped C3N4/ZnO nanoparticles for Ciprofloxacin removal: Degradation and mechanistic pathways, Int. J. Biol. Macromol. 198 (2022) 87–100. https://doi.org/10.1016/j.ijbiomac.2021.12.120.

[11] C. Du, J. Song, S. Tan, L. Yang, G. Yu, H. Chen, L. Zhou, Z. Zhang, Y. Zhang, Y. Su, X. Wen, S. Wang, Facile synthesis of Z-scheme ZnO/Ag/Ag3PO4 composite photocatalysts with enhanced performance for the degradation of ciprofloxacin, Mater. Chem. Phys. 260 (2021) 124136. https://doi.org/10.1016/j.matchemphys.2020.124136.
